# Integrative Analysis of Metallothioneins Identifies MT1H as Candidate Prognostic Biomarker in Hepatocellular Carcinoma

**DOI:** 10.3389/fmolb.2021.672416

**Published:** 2021-10-05

**Authors:** Feng Zhang, Shuijiao Guo, Wenhui Zhong, Kaijun Huang, Yubin Liu

**Affiliations:** ^1^ Department of General Surgery, Guangdong Provincial People’s Hospital, Guangdong Academy of Medical Sciences, Guangzhou, China; ^2^ Shantou University Medical College, Shantou, China; ^3^ Department of Operating Room, Guangdong Provincial People’s Hospital, Guangdong Academy of Medical Sciences, Guangzhou, China

**Keywords:** MT1H, hepatocellular carcinoma, metallothioneins, prognosis, biomarker

## Abstract

**Background:** Metallothioneins (MTs) play crucial roles in the modulation of zinc/copper homeostasis, regulation of neoplastic growth and proliferation, and protection against apoptosis. The present study attempted to visualize the prognostic landscape of MT functional isoforms and identify potential prognostic biomarkers in hepatocellular carcinoma (HCC).

**Methods:** The transcriptional expression, comprehensive prognostic performances, and gene–gene interaction network of MT isoforms in HCC were evaluated *via* Oncomine, GEPIA, Kaplan–Meier plotter, and GeneMANIA databases. Characterized by good prognostic value in three external cohorts, *MT1H* was specifically selected as a potential prognostic biomarker in HCC with various clinicopathological features. Functional and pathway enrichment analyses of *MT1H* status were performed using cBioPortal, the Database for Annotation, Visualization, and Integrated Discovery (DAVID), and ssGSVA method.

**Results:** *MT1E/1F/1G/1H/1M/1X/2A* was greatly downregulated in HCC. Prognostic analyses elucidated the essential correlations between *MT1A/1B/1H/1X/2A/4* attenuation and poor overall survival, between *MT1B/1H/4* downregulation and worse relapse-free survival, and between *MT1A/1B/1E/1H/1M/2A/4* downregulation and diminished progression-free survival in HCC. Taken together, these results indicated the powerful prognostic value of *MT1H* among MTs in HCC. In-depth analyses suggested that *MT1H* may be more applicable to alcohol-derived HCC and involved in the downregulation of the inflammatory pathway, Jak–STAT pathway, TNF pathway, and Wnt signaling pathway.

**Conclusion:** MT-specific isoforms displayed aberrant expression and varying prognostic value in HCC. *MT1H* repression in HCC was multi-dimensionally detrimental to patient outcomes. Therefore, *MT1H* was possibly associated with carcinogenesis and exploited as a novel prognostic biomarker and candidate therapeutic target for HCC.

## Introduction

Hepatocellular carcinoma (HCC) ranks as the second most lethal cancer accounting for almost 782,000 deaths annually (Bray et al., 2018). Despite therapeutic improvement, the prognosis of patients with HCC is unsatisfactory. Patients with early HCC who undergo surgical resection have an overall five-year survival rate of 50–70%, while patients with advanced HCC endure 25% one-year survival rate (European Association for the Study of the Liver, 2018). Discrepant therapeutic responses and undesirable prognoses may be attributed to the multi-task genomic variations, biological characteristics, and microenvironments even in the same focal region of HCC (Zhang et al., 2019). Due to distinct phenotypic and molecular heterogeneity in HCC, molecular-based prognosis prediction will shed light on recognition of its biological behaviors and support individual treatment allocation. However, emerging advances have not yet been clinically translated into useful prognostic biomarkers in HCC. Alpha-fetoprotein remains the most popular biomarker for predicting postoperative recurrence in patients with HCC, excluding those in Child–Pugh class A with cirrhosis and a single focal ≤3 cm undergoing radical resection (Chong et al., 2012; Giannini et al., 2012). Tremendous efforts to delineate molecular patterns associated with hepatocarcinogenesis are still being made to identify highly sensitive and specific biomarkers for prognosis assessment (Bell et al., 2011; Guan et al., 2019).

Metallothioneins (MTs) comprise an intracellular metal-binding protein family of 11 functional isoforms: *MT1* (*MT1A*, *MT1B*, *MT1E*, *MT1F*, *MT1G*, *MT1H*, *MT1M*, *MT1X*), *MT2A*, *MT3*, and *MT4* (Coyle et al., 2002), characterized by low molecular weight and high content of cysteine residues. In mammals, MTs play crucial roles in diversified processes, including manipulation of zinc/copper homeostasis, detoxification of heavy metals, regulation of cell growth and proliferation, and protection against oxidative stress and apoptosis (Si and Lang, 2018). Aberrant expression of MTs has been reported in multiple solid tumors, including HCC, some of which may be associated with prognosis. Decreased nuclear expression of *MT1* and *MT2* was an independent predictor and remarkably associated with high Edmondson–Steiner grade and micro-vascular invasion in HCC (Park and Yu, 2013). Serum *MT1M* and *MT1G* promoter methylation in HCC was greatly correlated with vascular invasion and distant metastases, serving as potential biomarkers (Ji et al., 2014; Zeng et al., 2018). Furthermore, *MT1H* is a member of the MT family and is abnormally expressed in malignancies. Previous studies elucidated that *MT1H* was downregulated in colorectal cancer (Jansová et al., 2006), osteosarcoma (Endo-Munoz et al., 2010), and prostate cancer (Han et al., 2013) and conversely upregulated in soft tissue sarcoma (Skubitz et al., 2012) and non-small-cell lung cancer (Werynska et al., 2013). Nevertheless, few studies have reported its prognostic role in HCC and relevant mechanisms. Few biological studies have been conducted on specific MT isoforms because of high sequence homology. Due to their isoform- and tissue-specific expression, systematic elucidation of the prognostic role and underlying mechanisms of individual MT isoforms in HCC is warranted.

To profile the role of specific MT isoforms and to identify promising biomarkers for HCC, here we evaluated the mRNA expression level of MTs between HCC and normal liver tissues *via* Oncomine and GEPIA databases and analyzed their prognostic value using the Kaplan–Meier plotter database, which were well validated in GSE54236, GSE116174, and ICGC-LIRI-JP cohorts. Further analyses of gene–gene interaction network and functional and pathway enrichment were conducted *via* GeneMANIA, cBioPortal, the DAVID, and ssGSVA method. Our results suggested that *MT1H* may serve as a novel candidate biomarker to illuminate prognosis prediction and potential therapeutic target in HCC.

## Materials and Methods

### Oncomine Database Analysis

The Oncomine database (https://www.oncomine.org /) is a web-based cancer microarray database that integrates 715 datasets, 18,761 microarrays, and 86,733 tissue samples, concentrating on the collection, standardization, and analysis of cancer transcriptional data (Rhodes et al., 2007). In the present study, we utilized it for evaluating MTs’ mRNA expression level in multiple cancers, especially in HCC. The search was conducted according to the following criteria: a) type of tissue: cancer vs. normal; b) data type: mRNA; and c) fold change>2 and *p* < 0.01.

### GEPIA Database Analysis

GEPIA (Gene Expression Profiling Interactive Analysis, http://gepia.cancer-pku.cn/) is an online interactive application tool for cancer and normal gene expressions on the basis of 9,736 tumors and 8,587 normal samples integrated from the TCGA and GTEx databases (Tang et al., 2017). Here, we used GEPIA for demonstrating the discrepancies of MTs’ mRNA expression between HCC tissue and normal hepatic tissue. *p* < 0.01 was considered statistically significant.

### Kaplan–Meier Plotter Analysis

As an online platform that facilitates the assessment of survival-related molecular biomarkers, the Kaplan–Meier plotter database (http://kmplot.com/) accommodates more than 10,000 cancer samples retrieved from public databases (Menyhárt et al., 2018). In this study, we used it to analyze the correlation between MTs’ expression and overall survival (OS), relapse-free survival (RFS), and progression-free survival (PFS) in HCC, based on hazard ratios (HRs) with 95% confidence intervals (CIs) and log-rank *p* values. *p* < 0.05 indicates statistical significance.

### TCGA and cBioPortal Analysis

The cBioPortal for Cancer Genomics (http://cbioportal.org/) embodies multifaceted data retrieved from The Cancer Genome Atlas (TCGA) database, including DNA copy-number alteration and methylation, mRNA and microRNA expressions, and protein (Gao et al., 2013). In this study, transcriptional expression data from the TCGA-HCC cohort were used to evaluate mutual correlations between MT family members.

### External Validation in GEO and ICGC Databases

To externally validate the prognostic performance of *MT1H* in HCC, publicly available transcriptional expression data and survival information of patients with HCC were collected from Gene Expression Omnibus (GEO, https://www.ncbi.nlm.nih.gov/geo/) and International Cancer Genome Consortium (ICGC, https://daco.icgc.org/) databases. Samples without complete survival information were removed from our study. Finally, GSE54236 (n = 81), GSE116174 (n = 64), and ICGC-LIRI-JP (n = 232) cohorts were included for independent validation.

### GeneMANIA Analysis

GeneMANIA (http://genemania.org/) provides an elastic interface accessible for gene function prediction, the discovery of genes with similar function, and the construction of gene function–related networks with regard to the given query gene list (Franz et al., 2018). In the present study, GeneMANIA was utilized to construct a gene–gene interaction network for the MT family in terms of shared protein domains, co-expression, prediction, co-localization, physical interactions, and pathways.

### Gene Function and Pathway Enrichment Analyses

According to Spearman’s correlation coefficient, co-expressed genes with MT1H were identified using the cBioPortal database. The Database for Annotation, Visualization, and Integrated Discovery (DAVID, https://david.ncifcrf.gov/) serves as a bioinformatics and analytic tool aimed at gene functional classification, chart, and table (Huang et al., 2009). Here, it was employed to perform Gene Ontology (GO) and Kyoto Encyclopedia of Genes and Genomes (KEGG) analyses with the purpose of enriched functional and biological pathway prediction of MT1H and its co-expressed genes.

### Gene Set Variation Analysis

GSVA was predominantly implemented to assign biologic and oncologic pathway activity between patients with high and low *MT1H* expressions (Hänzelmann et al., 2013). The gene sets of “c5.go.bp.v7.4.symbols.gmt” and “h.all.v7.4.symbols.gmt” were obtained from MSigDB (http://www.gsea-msigdb.org/gsea/msigdb/). Adjusted *p* values < 0.05 indicate statistical significance.

## Results

### mRNA Expression Level of MTs in Patients With HCC

**FIGURE 1:**
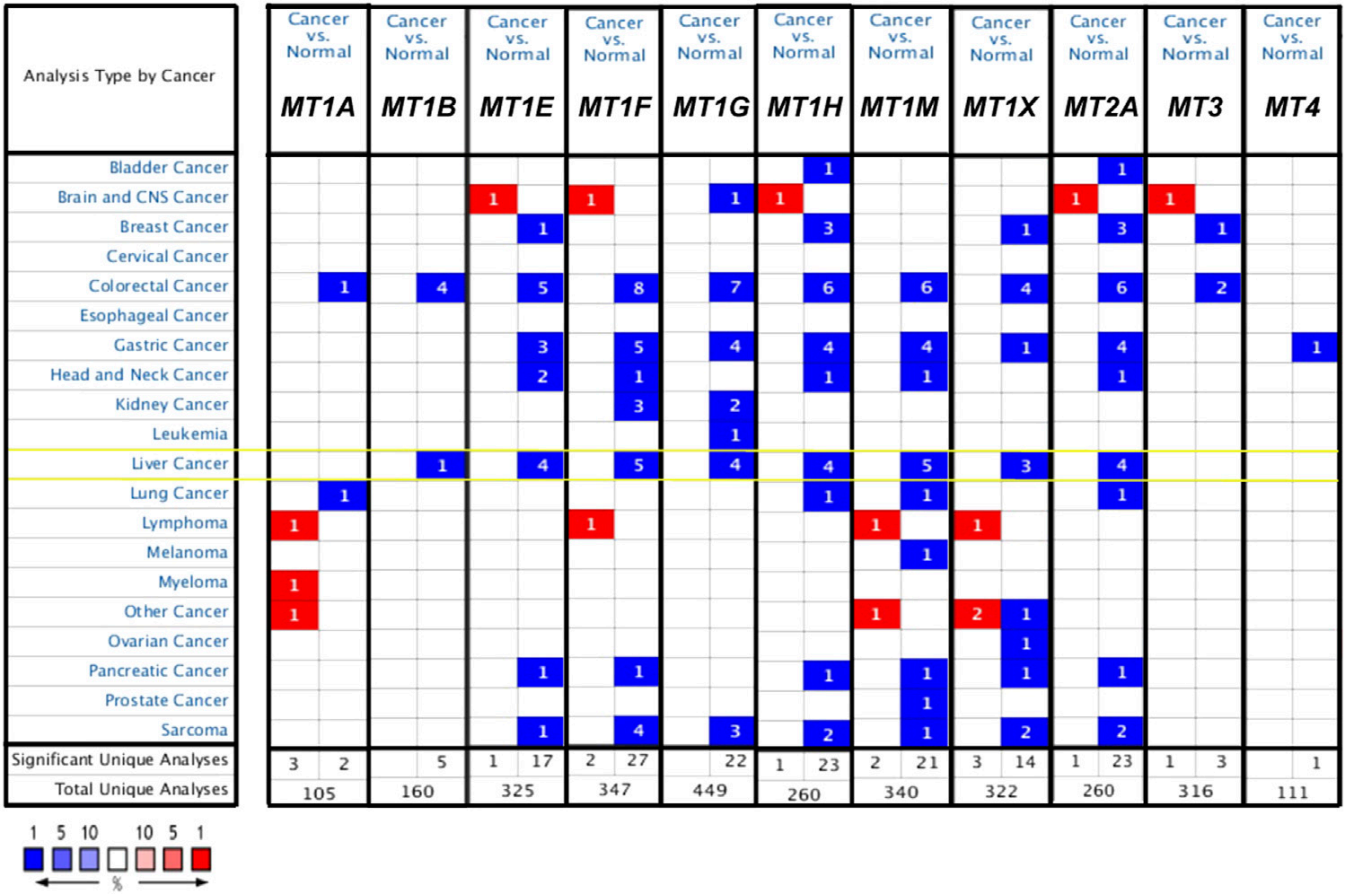
mRNA expression level of MTs between cancer and normal tumor tissues in multiple cancers from the Oncomine database. The numbers in the square represent the number of datasets that meet the threshold of fold change>2 and *p* < 0.01. The color intensity in red or blue is in direct proportion to the statistically significant level of *MT1H* upregulation or downregulation, respectively. Importantly, MTs’ expression changes in HCC are highlighted with yellow box.

The discrepancy of the transcriptional expression levels of 11 MT family members in multiple tumor tissue versus those in normal tissue was firstly analyzed using the Oncomine database (Figure 1 and Table 1). Stated roughly, Figure 1 shows an overview of MTs’ expression in 20 different cancer types. As shown in Table 1, most MTs’ mRNA expression was significantly downregulated in HCC. Chen et al. conducted a research study between 104 HCC cases and 76 normal liver cases and found that *MT1B* was transcriptionally diminished in HCC with fold change (FC) = −7.192 (Chen et al., 2002). Chen et al., Wurmbach et al., and Roessler et al. commonly described a lower cancerous expression of *MT1E* (FC = −8.957, −7.733, −8.999, respectively), *MT1F* (FC = −14.107, −9.680, −18.140, −15.749, respectively), *MT1G* (FC = −13.065, −11.187, −11.134, −11.160, respectively), *MT1H* (FC = −13.846, −7.723, −9.037, −8.473, respectively), and *MT1X* (FC = −10.812, −6.903, −11.558, −8.227, respectively) (Chen et al., 2002; Wurmbach et al., 2007; Roessler et al., 2010). Moreover, Roessler et al. also found that *MT1M* was statistically downregulated in HCC compared with normal liver tissue (FC = −17.300 and −28.314) (Roessler et al., 2010), similarly later reported by Mas et al. (FC = −8.149) (Mas et al., 2009). Wurmbach et al. and Roessler et al. had results essentially in agreement stating that *MT2A* was also lowly expressed in HCC versus normal liver tissue (FC = −4.402, −5.436, and −5.601) (Wurmbach et al., 2007; Roessler et al., 2010). Besides that, there were no available studies conducted for other MT isoforms that met the regulated criteria in the Oncomine database.

**TABLE 1:** Datasets of MTs in HCC from the Oncomine database.

MT	HCC (cases)	Normal (cases)	Fold change	*p*-Value	*t*-Test	Datasets/Refs.
*MT1A*	N/A	N/A	N/A	N/A	N/A	N/A
*MT1B*	HCC (104)	Liver (76)	−7.192	5.90E-36*	−15.996	Chen Chen et al, (2002)
*MT1E*	HCC (104)	Liver (76)	−8.957	2.96E-36*	−16.537	Chen Chen et al, (2002)
	HCC (35)	Liver (10)	−7.733	2.69E-11*	−8.677	Wurmbach Wurmbach et al, (2007)
	HCC (22)	Liver (21)	−8.999	7.53E-11*	−9.980	Roessler Roessler et al, (2010)
*MT1F*	HCC (103)	Liver (75)	−14.107	2.60E-42*	−18.769	Chen Chen et al, (2002)
	HCC (35)	Liver (10)	−9.680	2.00E-13*	−10.493	Wurmbach Wurmbach et al, (2007)
	HCC (22)	Liver (21)	−18.140	5.11E-16*	−12.724	Roessler Roessler et al, (2010)
	HCC (225)	Liver (220)	−15.749	2.12E-89*	−28.626	Roessler Roessler et al, (2010)
*MT1G*	HCC (104)	Liver (76)	−13.065	7.62E-37*	−16.102	Chen Chen et al, (2002)
	HCC (35)	Liver (10)	−11.187	4.01E-12*	−10.489	Wurmbach Wurmbach et al, (2007)
	HCC (22)	Liver (21)	−11.134	9.25E-13*	−10.810	Roessler Roessler et al, (2010)
	HCC (225)	Liver (220)	−11.160	1.20E-74*	−25.426	Roessler Roessler et al, (2010)
*MT1H*	HCC (104)	Liver (75)	−13.846	1.85E-40*	−17.875	Chen Chen et al, (2002)
	HCC (35)	Liver (10)	−7.723	1.13E-12*	−9.861	Wurmbach Wurmbach et al, (2007)
	HCC (22)	Liver (21)	−9.037	5.37E-11*	−10.079	Roessler Roessler et al, (2010)
	HCC (225)	Liver (220)	−8.473	5.26E-70*	−24.361	Roessler Roessler et al, (2010)
*MT1M*	HCC (225)	Liver (220)	−17.300	1.49E-86*	−25.903	Roessler Roessler et al, (2010)
	HCC (22)	Liver (21)	−28.314	1.84E-13*	−12.149	Roessler Roessler et al, (2010)
	HCC (38)	Liver (19)	−8.149	7.49E-11*	−7.919	Mas Mas et al, (2009)
*MT1X*	HCC (104)	Liver (76)	−10.812	7.56E-34*	−15.462	Chen Chen et al, (2002)
	HCC (35)	Liver (10)	−6.903	2.54E-12*	−9.488	Wurmbach Wurmbach et al, (2007)
	HCC (22)	Liver (21)	−11.558	2.41E-12*	−10.894	Roessler Roessler et al, (2010)
	HCC (225)	Liver (220)	−8.227	1.05E-70*	−24.663	Roessler Roessler et al, (2010)
*MT2A*	HCC (35)	Liver (10)	−4.402	7.87E-12*	−9.089	Wurmbach Wurmbach et al, (2007)
	HCC (22)	Liver (21)	−5.436	3.14E-11*	−10.099	Roessler Roessler et al, (2010)
	HCC (225)	Liver (220)	−5.601	2.72E-66*	−23.883	Roessler Roessler et al, (2010)
*MT3*	N/A	N/A	N/A	N/A	N/A	N/A
*MT4*	N/A	N/A	N/A	N/A	N/A	N/A

**FIGURE 2:**
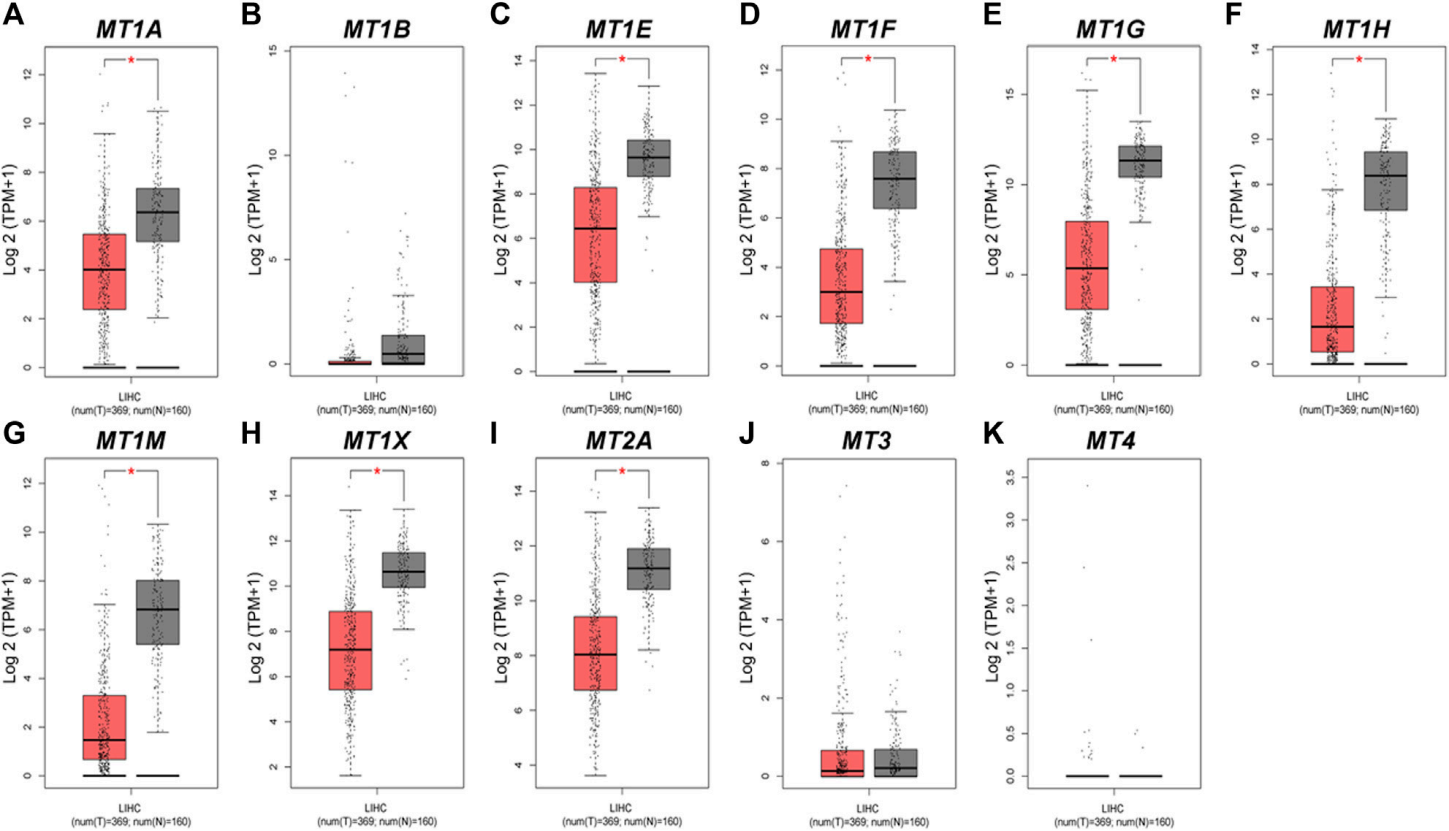
Transcriptional expression levels of MTs in HCC and normal liver tissues from the GEPIA database. **(A–K)** Expressional comparison of 11 MT family members in HCC (red plot) and normal (gray plot) tissues. *p < 0.01.

Moreover, we evaluated the differential mRNA expression levels of MTs between HCC and normal hepatic tissues *via* the GEPIA database (Figure 2). Suppression of *MT1E*, *MT1F*, *MT1G*, *MT1H*, *MT1M*, *MT1X*, and *MT2A* expressions was found to be significant in HCC (Figures 2C–I, *p* < 0.01), while *MT3* and *MT4* had no differential mRNA expression, in line with the results analyzed by the Oncomine database (Figures 2J,K). Meanwhile, although lower expression of *MT1A* and no differential expression of *MT1B* were found in GEPIA (Figures 2A,B), the results from the Oncomine database were not consistent with them. Taken together, Oncomine and GEPIA databases jointly suggested that the mRNA expression of *MT1E*, *MT1F*, *MT1G*, *MT1H*, *MT1M*, *MT1X*, and *MT2A* was significantly downregulated in patients with HCC.

### MTs Displayed Varying Prognostic Significance in Patients With HCC

**FIGURE 3:**
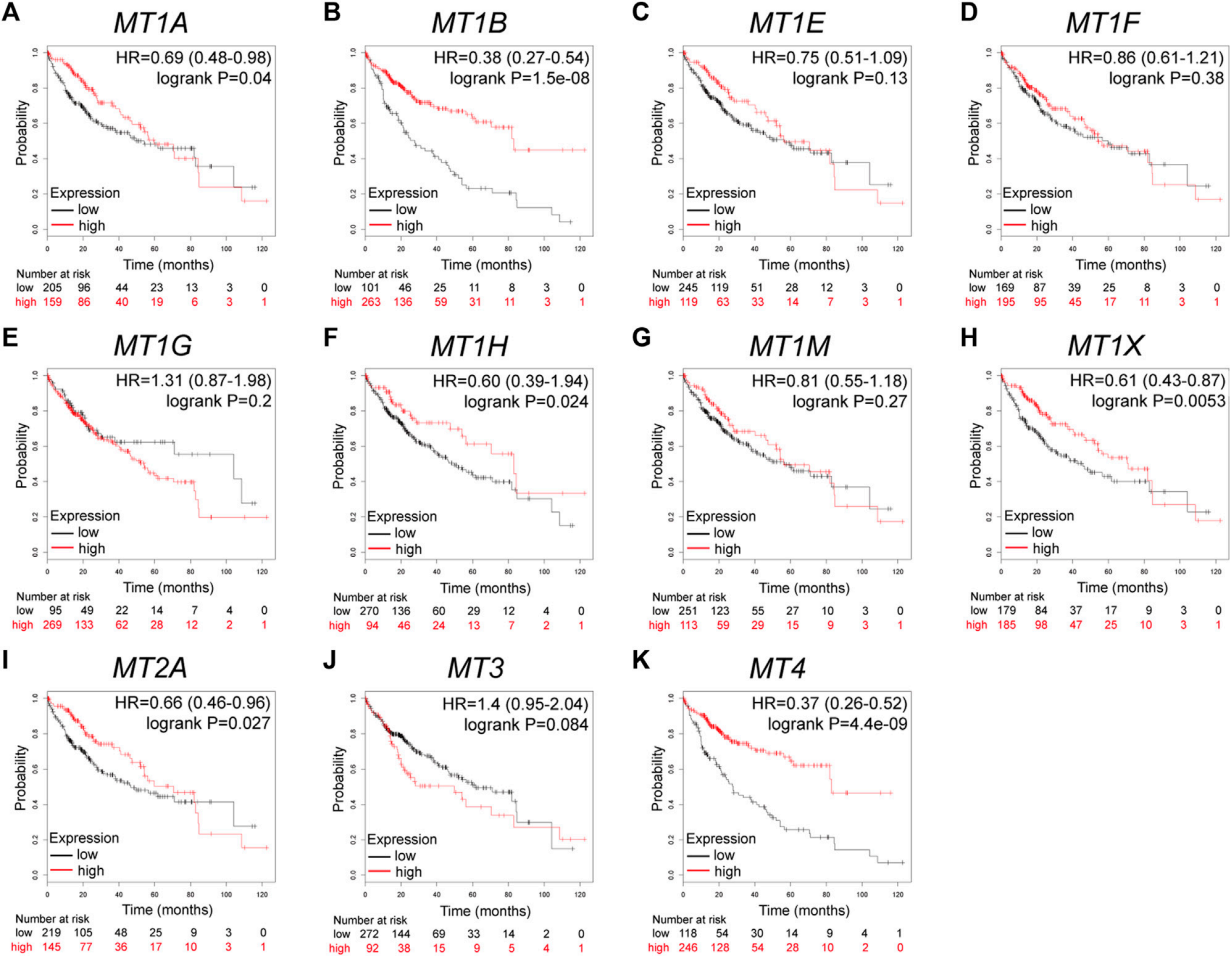
Prognostic values of specific MT isoforms in HCC: **(A–K)** OS in Kaplan–Meier plotter. The *p*-values were calculated by the log-rank test. OS, overall survival.

**FIGURE 4:**
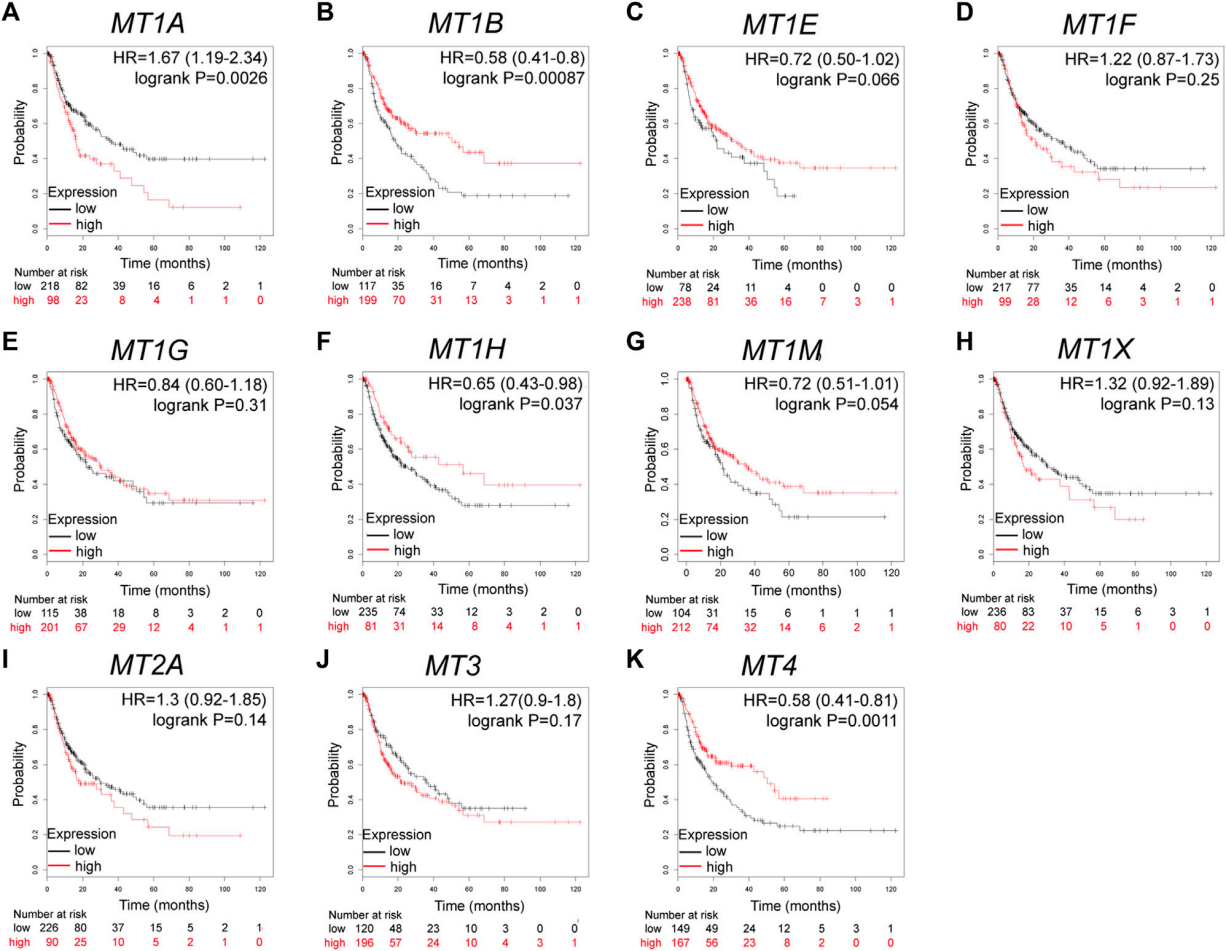
Prognostic values of specific MT isoforms in HCC: **(A–K)** RFS in Kaplan–Meier plotter. The *p*-values were calculated by the log-rank test. RFS, recurrence-free survival.

**FIGURE 5:**
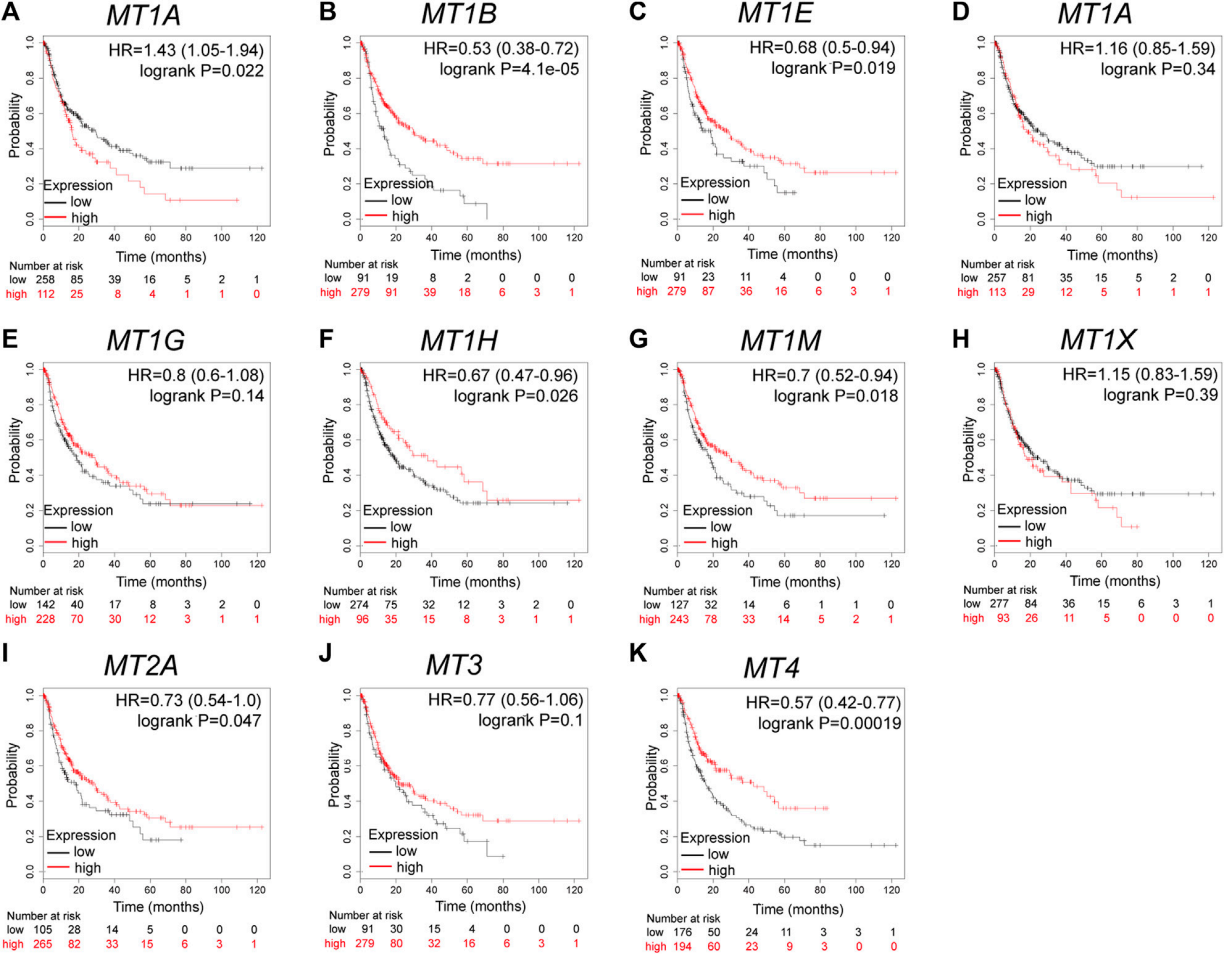
Prognostic values of specific MT isoforms in HCC: **(A–K)** PFS in Kaplan–Meier plotter. The *p*-values were calculated by the log-rank test. PFS, progression-free survival.

Further analysis using the Kaplan–Meier plotter database displayed the prognostic values of MT family members in overall survival (OS, Figures 3A–K), recurrence-free survival (RS, Figures 4A–K), and progression-free survival (PFS, Figures 5A–K). What stood out in the results was that downregulation of *MT1A*, *MT1B*, *MT1H*, *MT1X*, *MT2A*, and *MT4* was significantly correlated with poor OS in patients with HCC (Figures 3A,B,F,H,I,K). Suppressed *MT1B*, *MT1H*, and *MT4* expressions were closely related to worse RFS in HCC (Figures 4B,F,K). Then, Figure 5 shows the correlation of lower *MT1B*, *MT1E*, *MT1H*, *MT1M*, *MT2A*, and *MT4* mRNA expressions with poor PFS (Figures 5B,C,F,G,I,K). Interestingly, MT1A showed the protective effect on OS (HR = 0.69, Figure 3A) and, oppositely, the disruptive effect on RFS (HR = 1.67, Figure 4A) and PFS (HR = 1.43, Figure 5A). In total, MT family members displayed various prognostic values, among which *MT1B*, *MT1H*, and *MT4* were consistently correlated with OS, RFS, and PFS in patients with HCC. As *MT1H* was transcriptionally downregulated and displayed great prognostic value, we considered *MT1H* as a potential prognostic biomarker in HCC from the MT family and searched for in-depth evaluation.

### Correlation of *MT1H* Expression and Prognosis in Stratified Patients With HCC and Different Clinicopathological Factors

We investigated the relationship between *MT1H* expression and prognosis in patients with HCC and diverse clinicopathological features by the Kaplan–Meier plotter database. Table 2 illustrates that *MT1H* exhibited a protective effect on OS and PFS in patients with the following characteristics: male (OS: HR = 0.52, *p* = 0.03; PFS: HR = 0.65, *p* = 0.043), white (OS: HR = 0.42, *p* = 0.008; PFS: HR = 0.54, *p* = 0.01), TNM stage 3 (OS: HR = 0.47, *p* = 0.048; PFS: HR = 0.5, *p* = 0.029), TNM stage 3 + 4 (OS: HR = 0.45, *p* = 0.038; PFS: HR = 0.49, *p* = 0.022), grade 1 (OS: HR = 0.29, *p* = 0.046; PFS: HR = 0.23, *p* = 0.003), AJCC-T 3 (OS: HR = 0.42, *p* = 0.042; PFS: HR = 0.45, *p* = 0.012), and alcohol consumption (OS: HR = 0.32, *p* = 0.0033; PFS: HR = 0.45, *p* = 0.01). Totally, these results indicated that the prognostic values of *MT1H* expression in HCC were based on distinctive clinicopathological factors and mostly applicable to male patients with advanced TNM or AJCC-T stage, with well-differentiated tumor and with alcohol consumption.

**TABLE 2:** *MT1H* expression and prognosis in HCC with different clinicopathological factors.

Clinicopathological factors	Overall survival (OS)			Progression-free survival (PFS)		
	N	HR	P	N	HR	P
Sex						
Female	118	1.43 (0.82–2.51)	0.21	120	1.35 (0.78–2.32)	0.28
Male	246	0.52 (0.29–0.95)	**0.03**	246	0.65 (0.42–0.99)	**0.043**
Race						
White	181	0.42 (0.21–0.81)	**0.008**	183	0.54 (0.34–0.87)	**0.01**
Asian	155	0.55 (0.26–1.19)	0.12	155	0.7 (0.4–1.23)	0.21
TNM stage						
1	170	0.75 (0.37–1.53)	0.42	170	1.52 (0.86–2.68)	0.15
2	83	3.03 (0.9–10.24)	0.062	84	0.64 (0.35–1.19)	0.15
**3**	83	0.47 (0.22–1.01)	**0.048**	83	0.5 (0.27–0.94)	**0.029**
4	4	—	—	5	—	—
1 + 2	253	1.36 (084–2.21)	0.21	254	0.72 (0.45–1.16)	0.17
3 + 4	87	0.45 (0.21–0.98)	**0.038**	88	0.49 (0.26–0.91)	**0.022**
Grade						
**1**	55	0.29 (0.08–1.04)	**0.046**	55	0.23 (0.08–0.64)	**0.003**
2	174	3.21 (1.5–6.87)	**0.0016**	175	1.51 (0.95–2.41)	0.076
3	118	0.61 (0.29–1.2)	0.18	119	0.74 (0.42–1.31)	0.3
4	12	—	—	12	—	—
AJCC-T						
1	180	0.74 (0.38–1.47)	0.39	180	1.51 (0.87–2.61)	0.14
2	90	2.29 (0.87–6.06)	0.085	92	0.55 (0.3–1.01)	0.05
**3**	78	0.42 (0.18–0.99)	**0.042**	78	0.45 (0.23–0.85)	**0.012**
4	13	—	—	13	—	—
Vascular invasion						
Yes	90	3.09 (1.06–9.03)	**0.03**	91	0.67 (0.34–1.32)	0.24
None	203	0.55 (0.31–0.98)	**0.04**	204	0.63 (0.38–1.06)	0.079
Alcohol consumption						
Yes	115	0.32 (0.14–0.71)	**0.0033**	115	0.45 (0.24–0.84)	**0.01**
None	202	0.74 (0.42–1.31)	0.3	204	0.77 (0.48–1.25)	0.29
Virus hepatitis						
Yes	150	1.5 (0.78–2.88)	0.22	152	0.67 (0.42–1.07)	0.09
None	167	0.34 (0.17–0.66)	**8e-04**	167	0.62 (0.37–1.03)	0.061

* P value <0.05 was defined as significant and marked in bold

### External Validation, Mutual Correlations, and Gene–Gene Interaction Network of MTs in HCC

**FIGURE 6:**
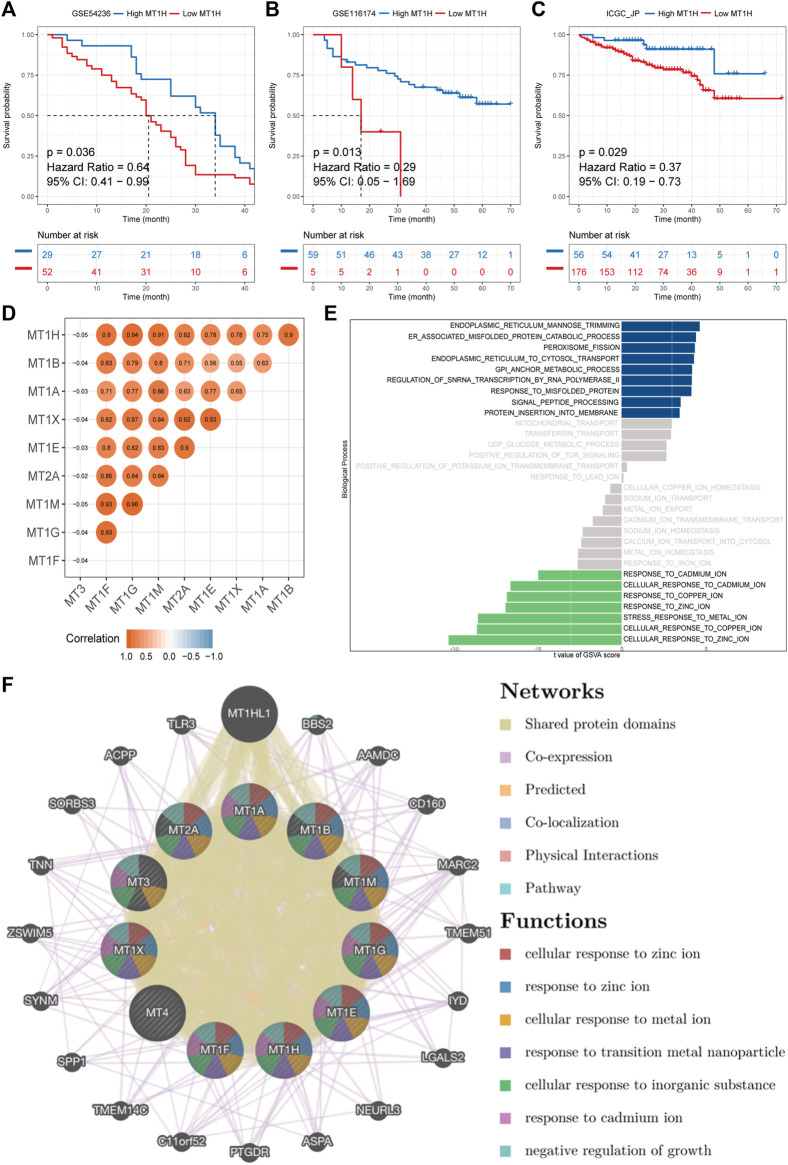
External validation, mutual correlations, and gene–gene interaction network of MTs in HCC. **(A–C)** Kaplan–Meier survival analyses of *MT1H* expression in GSE54236, GSE116174, and ICGC-LIRI-JP cohorts externally validate the prognostic value of *MT1H*. **(D)** The mutual correlation matrix shows positive Spearman’s correlation of MT family members. **(E)** ssGSVA on the biological process between samples with high or low *MT1H* expression. **(F)** Gene–gene interaction network among MT isoforms. Each node represents one MT family member. The size and color of nodes represent the strength of interactions and genetic functions, while the line color represents the types of gene–gene interactions.

To validate the prognostic value and robustness of *MT1H* in HCC, its performance was evaluated in three independent cohorts, including GSE54236, GSE116174, and ICGC-LIRI-JP. The optimal cut-off values of MT1H expression in the cohorts were defined by the “surv_cutpoint” function from the “survminer” R package for significant continuous variables. We found that *MT1H* also externally worked well, where patients with low *MT1H* expression had unfavorable OS (GSE54236, HR = 0.64, *p* = 0.036, Figure 6A; GSE116174, HR = 0.29, *p* = 0.013, Figure 6B; ICGC-LIRI-JP, HR = 0.37, *p* = 0.029, Figure 6C). Then, we acquired complete MTs’ mRNA expression data in HCC apart from *MT4* expression not available to evaluate the correlations between MTs in HCC. The results indicated statistically significant positive correlations among *MT1A*, *MT1B*, *MT1E*, *MT1F*, *MT1G*, *MT1H*, *MT1M*, *MT1X*, and *MT2A*, while no significant correlation was found between *MT3* and other MT family members (Figure 6D).

Furthermore, ssGSVA was performed to reveal biological function enrichment on different *MT1H* expression statuses. Patients with lower *MT1H* expression were characterized by impaired zinc, copper, and cadmium ion activity (Figure 6E). A functional-associated gene–gene interaction network of MTs was constructed using the GeneMANIA database (Figure 6F and Supplementary Material S2). MTs were surrounded by 20 nodes representing genes that closely associated with MTs in terms of 52.16% shared protein domains, 36.66% co-expression, 10.91% prediction, and 0.27% co-localization. All MT proteins had shared protein domains with each other and particularly with *MT1HL1* (metallothionein 1H–like 1), which exhibited the greatest correlation with the MT family and was co-expressed with *MT1E*, *MT1F*, *MT1G*, *MT1H*, *MT1M*, *MT1X*, and *MT2A*. Apart from *MT1HL1*, the top two genes significantly correlated with the MT family were *BBS2* (Bardet–Biedl syndrome 2) and *AAMDC* (adipogenesis associated, Mth938 domain containing). Further functional-associated analysis revealed that the MT family performed essential functions in response to zinc, metal, and cadmium ions, response to transition metal nanoparticles, cellular response to inorganic substances, and negative regulation of growth (Supplementary Material S2).

### Functional and Pathway Enrichment Analyses of *MT1H* and Its Co-Expressed Genes

**FIGURE 7:**
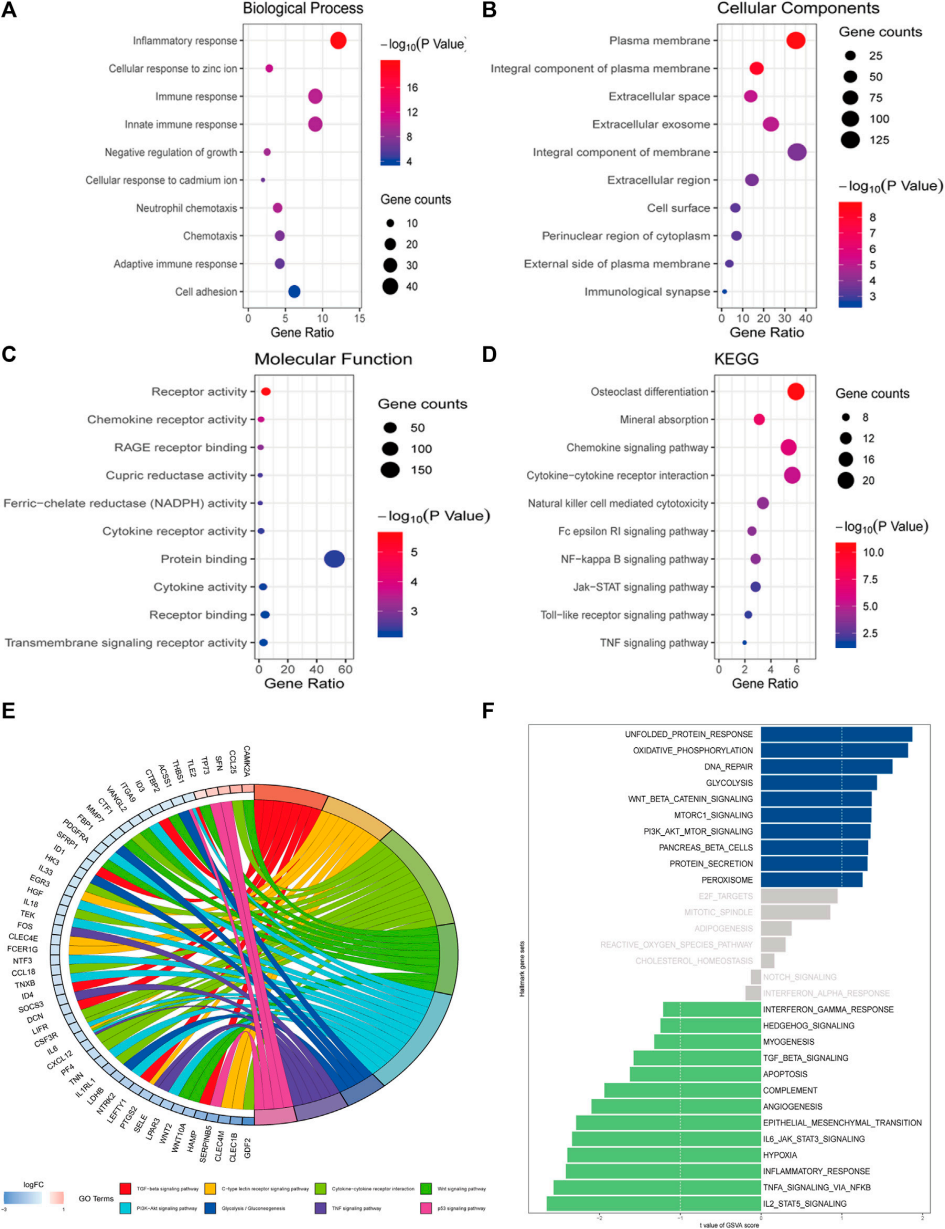
Functional and pathway enrichment analyses of *MT1H* and its co-expressed genes. **(A–D)** Bubble plots representing enriched functions and pathways of genes co-expressed with MT1H. **(E)** Analysis on the KEGG gene set between samples with high or low MT1H expression. **(F)** ssGSVA on the hallmark gene set between samples with high or low *MT1H* expression.

Obtained from the cBioPortal database with Spearman’s correlation coefficient exceeding 0.25, a query list of 358 genes co-expressed with *MT1H* was adopted for GO/KEGG gene function and pathway enrichment analyses using the DAVID (Figures 7A–D). Bubble plots for GO enrichment analysis demonstrated functional forecast of the given genes in terms of biological processes, cellular components, and molecular functions. The results were found to be significant stating that *MT1H* and its co-expressed genes were associated with GO: 0071294 (cellular response to zinc ions), GO: 0045926 (negative regulation of growth), GO: 0071276 (cellular response to cadmium ions), and GO: 0007155 (cell adhesion), essential for metal carcinogenesis, cell proliferation, and cellular differentiation (Figure 7A). Intriguingly, KEGG enrichment analysis defined functional-associated pathways of the genes co-expressed with *MT1H*, among which hsa04064 (NF-kappa B signaling pathway), hsa04630 (Jak–STAT signaling pathway), hsa04620 (Toll-like receptor signaling pathway), and hsa04668 (TNF signaling pathway) were involved in tumor growth and progression (Figure 7D).

To assign pathway activity between different *MT1H* expression patterns and illuminate the intrinsic mechanism, we performed KEGG analysis and ssGSVA between samples with high or low *MT1H*. Downregulation of the TGF-beta signaling pathway, Wnt signaling pathway, PI3K−Akt signaling pathway, TNF signaling pathway, and p53 signaling pathway involved in *MT1H*-related carcinogenesis (Figure 7E). Analysis on hallmark gene sets also revealed that samples with low *MT1H* expression were marked by decreased TGF-beta signaling pathway, Jak–STAT pathway, inflammatory response pathway, and TNF signaling pathway (Figure 7F).

## Discussion

Tumor prognostication assessment refers to the prediction of the likelihood of cancerous outcomes. Good outcomes conventionally include remission, amelioration, and stabilization, whereas poor outcomes represent relapse, progression, and mortality. Prognostic biomarkers act as powerful tools for reflecting life expectancy in patient management, and they are critical in guiding clinical oncology staging derived from common prognostic properties and distinguishing survival benefit in clinical trials (Nault and Villanueva, 2020).

Great efforts have been made in the past few decades to search for candidate prognostic biomarkers in HCC, among which the prognostic significance of MTs has been preliminary elucidated. Lower nuclear expression of *MT1* and *MT2* was associated with well-differentiated grade, macro-vascular invasion, and poor long-term survival in patients with HCC (Park and Yu, 2013; Yang et al., 2018; Wang and Gribskov, 2019; Tamai et al., 2020). In addition, *MT1* was identified as a potential biomarker indicating the effect of sorafenib on the redox metabolism in HCC cells (Houessinon et al., 2016). However, despite the noted discrepancies between MT isoforms like affinity to metal iron and susceptibility to antioxidants, these differences cannot justify 11 MT functional isoforms, indicating that some profound biological significance remains to be investigated (Krizkova et al., 2018). Moreover, the contribution of individual MT isoforms to the overall prognostic value is still poorly understood. To the best of our knowledge, the present study is the first attempt to link clinical outcomes with all MT isoforms in HCC and identify particular isoforms that account for the predominant prognostic value of the MT family. Here, we attempted to use a bioinformatics approach for biomarker investigation, and our results confirmed that MT isoforms displayed unequal outcome-prediction values and that *MT1H* is a potential novel prognostic biomarker in HCC.

Our results confirmed the significant downregulation of *MT1H* in HCC and its significant role in hepatic carcinogenesis and progression. Most importantly, the powerful prognostic significance of *MT1H*, which greatly correlated with OS, PFS, and RFS in patients with HCC, was demonstrated for the first time. The prognostic performance of *MT1H* was well validated in three independent cohorts. *MT1H* was extremely important in evaluating the survival and prognosis in patients with advanced TNM or AJCC-T stage, well-differentiated tumor, and alcohol consumption.

Chronic alcohol consumption is one of the well-known etiological factors of HCC, and growing evidence suggests the indispensable status of epigenetic features in alcohol-mediated carcinogenesis (Seitz and Stickel, 2007). Ethanol had an effect on one-carbon metabolism and accelerated tumor spread through the alteration of methyl groups required for methylation events, including those of DNA and histone. MTs also participated in the abovementioned process. *ZNF479*-induced *MT1* transcriptional repression was restored by silencing DNA methyltransferase 1 (*DHMT1*), suggesting potential therapeutic strategies for HCC that target the *ZNF479/MLL* complex/*MT1* or related epigenetic factors (Wu et al., 2019). The promoter region of *MT1H* was hypermethylated in cancer, and conversely, demethylation of the *MT1H* promoter reversed the suppression of *MT1H* expression (Gong et al., 2019). Promoter hypermethylation–derived transcriptional inhibition of tumor suppressor genes may turn into biomarkers in the progression of liver cancer (Hardy and Mann, 2016). Acting as a hypermethylated gene and implicating an epigenetic property of alcohol-associated HCC, *MT1H* has already exhibited its latent capacity of turning into epigenetic markers for this subtype of neoplasia (Udali et al., 2015). Moreover, it has been claimed that the tumor suppressor activity of *MT1H* depends on its ability to interact and to promote histone methyltransferase activity of euchromatin histone methyltransferase 1 (*EHMT1*) involved in the carcinogenesis of prostate cancer, which remains to be discussed in those with HCC (Han et al., 2013). In summary, these studies highlight the potential effect of epigenetic modification involved in prognostication assessment of *MT1H*, particularly in alcohol-associated HCC.

Using the GeneMANIA database, GO analysis, and ssGSVA, functional analysis of *MT1H* and its co-expressed genes indicated their association with cellular response to zinc and cadmium ions and negative regulation of growth. Cytoplasmic MTs reached maximal value in the presence of zinc during the G1/S phase transition, pointing to a regulatory role of MTs in tumor cell proliferation that modulated the zinc level and the activity of zinc-dependent transcriptional factor (Nagel and Vallee, 1995). Intriguingly, decreased expression of *MT1H* impaired the colony formation and interfered with prostate cancer and HCC cells entering the S and M cell cycle phases to suppress cell growth (Han et al., 2013; Zheng et al., 2017). Thus, we hypothesize that *MT1H* may make a crucial contribution to tumor growth by governing the supply of zinc during a period when cells get ready to undergo DNA synthesis.

Pathway analyses on both KEGG and hallmark gene sets revealed the regulation of *MT1H* expression through the downregulation of several pathways that participated in carcinogenesis, such as inflammatory-related pathway-like NF-κB pathway, Jak–STAT pathway, TNF pathway, and Wnt signaling pathway. Greater than 90% of HCC is inflammation-initiated and commonly derived from chronic HBV or HCV infection regulated by the NF-κB signaling pathway that orchestrates the manufacturing of inflammatory mediators such as tumor necrosis factor α (*TNFα*) (Yu et al., 2018). However, recent studies unmasked some paradoxical effects of NF-κB signaling pathway in HCC. For example, inactivation of the NF-κB pathway controlled by conditional deletion of IκB kinase β in the liver promoted chemical carcinogenesis of HCC (Maeda et al., 2005). A similar situation also exists in the investigation of MT impact on hepatocarcinogenesis. Downregulation of *MT1M* increased the activity of NF-κB, which resulted in the inhibition of apoptosis and contributed to hepatocarcinogenesis (Mao et al., 2012). In contrast, MT’s overexpression and its autophagic lysosomal relocation greatly impaired the toxic iron-centered oxidative stress caused by TNF in HTC cells (Ullio et al., 2016). Our present results also showed that *MT1H* was probably associated with NF-κB and *TNF* pathways. These studies indicated that more knowledge of *MT1H*-regulated NF-κB and *TNF* pathways in combination with related regulatory mechanisms may provide a favorable strategy for HCC management. Furthermore, accumulating *in vivo* and *in vitro* studies have indicated the key role of the Jak–STAT signaling pathway implicated in MTs’ protection in doxorubicin-mediated cardiomyopathy and astrocytic reactions to traumatic brain injury (Leung et al., 2010; Shuai et al., 2011). Inactivated Jak–STAT signaling pathway may serve as an effective therapeutic strategy in growth inhibition of SP/CD44 + tumorigenic cells in Akt/β-catenin–driven HCC (Toh et al., 2020). Intriguingly, MT1H inhibited the Wnt/β-catenin signal transduction pathway *via* the Akt/Gsk-3β axis, thus suppressing proliferation, invasion, and migration of HCC cells (Zheng et al., 2017). Therefore, delineating the link between *MT1H* and HCC through modulating the Jak–STAT pathway at the crossroad of Akt/mTOR and Wnt/β-catenin pathways may provide new insights into a promising therapeutic approach.

*MT1X* and *MT2A* are regarded as two prominent molecules associated with carcinogenesis and progression that have previously not attracted enough attention. *MT1X* was upregulated in the human hepatoma cell line HepG2 with acute exposure to cadmium chloride, whose major toxicological effects were liver hyperplasia, liver tumor, liver degeneration, fibrosis, and cirrhosis (Cartularo et al., 2015). Affected by cadmium-induced DNA damage, the tumor suppressor p53 protein appeared to inactivate and suppress mediated cell cycle arrest (Fabbri et al., 2012). Overexpression of *MT1X* induced cell cycle arrest and promoted apoptosis by inhibiting the NF-кB signaling pathway in HCC (Liu et al., 2018). In addition, *XAF1*-mediated *MT2A* inactivation resulted in higher free intracellular zinc levels and p53 upregulation. Through the antagonistic effect of *MT2A*, *XAF1* played a crucial role in apoptosis promotion and cellular fateful decisions under conditions of stress caused by zinc and other metals (Shin et al., 2017). MTs could transfer zinc ions from the p53 protein, causing mutation-like spatial structural changes and functional loss, ultimately contributing to uncontrolled cellular proliferation. Thus, the interaction of MTs such as *MT1X* and *MT2A* with the p53 protein seems to be crucial for carcinogenesis and progression (CARDOSO et al., 2009). Our results also showed that *MT1X* and *MT2A* were lowly expressed and significantly correlated with poor OS in patients with HCC, essential in response to cadmium ions and negative regulation of growth. Hence, we speculate that *MT1X* and *MT2A* participate in the carcinogenesis and progression of HCC through modulating the p53 protein and NF-κB signaling pathway when acutely exposed to cadmium. In our study, double-edged prognostic effects of *MT1A* were firstly discovered. *MT1A* expression was correlated with favorable OS and, on the contrary, with worse RFS and PFS. The intrahepatic copper concentrations elevate as the first liver’s response toward *MT1A* overexpression. In livers with copper-associated hepatitis induced by *MT1H*, activation of inflammatory pathways could be detected. Later in the chronic transformation of liver inflammation, changes are found in cell adhesion adaptations and cytoskeleton remodeling (Dirksen et al., 2017). The current studies about *MT1A* could not accurately explain its opposite prognostic effects, leaving broadened scope for future research.

There are some limitations to our study. First, a high rate of analyzed sequencing and microarray data was integrated from diverse bioinformatics databases or platforms, which may lead to background heterogeneity to some extent. Second, although we demonstrated the prognostic value of *MT1H* for HCC in multiple dimensions, further validation is necessary because a scientifically rigorous approach for biomarker validation following the predefined stages of biomarker excavation will enhance clinical validity of biomarkers in oncology management (Pepe et al., 2008). Third, although *MT1H* is effective in the preliminary investigation, it is limited as a single molecular biomarker without combination with other molecules, imaging, or clinical parameters to maximize its performance (Chaiteerakij et al., 2015).

In conclusion, this comprehensive study of prognostic biomarker development using in silico approaches elucidated that most MT functional members are uniformly suppressed in HCC and contribute to hepatocarcinogenesis and progression. Repressed *MT1H* expression is greatly associated with poor survival in patients with HCC and particularly in alcohol-related HCC. *MT1H* may function in regulating cellular response to zinc ions and negatively modulate tumor growth *via* the downregulation of the inflammatory pathway, Jak–STAT pathway, TNF pathway, and Wnt signaling pathway. Based on these findings, we believe that *MT1H* could be a qualified candidate to become a novel prognostic biomarker in HCC.

## Data Availability

The data that support our results and findings are available from the databases: The Oncomine (https://www.oncomine.org/), GEPIA (http://gepia.cancer-pku.cn/), Kaplan-Meier plotter (http://kmplot.com/), cBioPortal (http://cbioportal.org/), GEO (https://www.ncbi.nlm.nih.gov/geo/), ICGC (https://daco.icgc.org/), GeneMANIA (http://genemania.org/), DAVID (https://david.ncifcrf.gov/) and MSigDB (http://www.gsea-msigdb.org/gsea/msigdb/).
