# Supplementary material for: Integrative Analysis of Metallothioneins Identifies MT1H as Candidate Prognostic Biomarker in Hepatocellular Carcinoma
Source: Front Mol Biosci. 2021 Oct 5;8:672416. doi: 10.3389/fmolb.2021.672416 (PMC8523949; doi:10.3389/fmolb.2021.672416)
Supplement: Supplementary file 1 [file DataSheet2.PDF]

Function	FDR	Genes in network	Genes in genome
cellular response to zinc ion	3.39e-21	9	10
response to zinc ion	8.44e-19	9	15
cellular response to metal ion	6.01e-16	10	48
response to transition metal nanoparticle	6.01e-16	9	28
cellular response to inorganic substance	1.16e-15	10	52
response to cadmium ion	1.29e-14	7	11
negative regulation of growth	1.39e-13	11	129
response to metal ion	1.03e-12	10	103
response to inorganic substance	5.29e-11	10	153
regulation of growth	6.08e-10	11	283
zinc ion binding	2.10e-9	10	225
perinuclear region of cytoplasm	1.47e-8	10	276
