# Supplementary material for: Integrative Analysis of Metallothioneins Identifies MT1H as Candidate Prognostic Biomarker in Hepatocellular Carcinoma
Source: Front Mol Biosci. 2021 Oct 5;8:672416. doi: 10.3389/fmolb.2021.672416 (PMC8523949; doi:10.3389/fmolb.2021.672416)
Supplement: Supplementary file 2 [file DataSheet1.PDF]

Organism: H. sapiens
Application version: 3.6.0
Database version: 13 March 2017 00:00:00
Network generated on: 31 January 2020
Author: GeneMANIA (genemania.org)
Notes: Network weight reflects the data source relevance for predicting the function of interest

Entity 1	Entity 2	Weight	Network group	Network
MT1B	MT1A	0.05423763	Co-expression	Noble-Diehl-2008
MT1M	MT1A	0.046342306	Co-expression	Noble-Diehl-2008
MT1M	MT1B	0.05180134	Co-expression	Noble-Diehl-2008
MT1G	MT1A	0.052168403	Co-expression	Noble-Diehl-2008
MT1G	MT1B	0.05728338	Co-expression	Noble-Diehl-2008
MT1G	MT1M	0.048709396	Co-expression	Noble-Diehl-2008
MT1E	MT1A	0.04045814	Co-expression	Noble-Diehl-2008
MT1E	MT1B	0.044736736	Co-expression	Noble-Diehl-2008
MT1E	MT1M	0.038894396	Co-expression	Noble-Diehl-2008
MT1E	MT1G	0.042798847	Co-expression	Noble-Diehl-2008
MT1H	MT1A	0.054227617	Co-expression	Noble-Diehl-2008
MT1H	MT1B	0.058949064	Co-expression	Noble-Diehl-2008
MT1H	MT1M	0.05041264	Co-expression	Noble-Diehl-2008
MT1H	MT1G	0.05670659	Co-expression	Noble-Diehl-2008
MT1H	MT1E	0.043866623	Co-expression	Noble-Diehl-2008
MT1F	MT1A	0.04561124	Co-expression	Noble-Diehl-2008
MT1F	MT1B	0.051812503	Co-expression	Noble-Diehl-2008
MT1F	MT1M	0.048775434	Co-expression	Noble-Diehl-2008
MT1F	MT1G	0.05082554	Co-expression	Noble-Diehl-2008
MT1F	MT1E	0.038008593	Co-expression	Noble-Diehl-2008
MT1F	MT1H	0.051114902	Co-expression	Noble-Diehl-2008
MT4	MT1A	0.028839478	Co-expression	Noble-Diehl-2008
MT4	MT1B	0.026561704	Co-expression	Noble-Diehl-2008
MT4	MT1M	0.018332558	Co-expression	Noble-Diehl-2008
MT4	MT1G	0.02509931	Co-expression	Noble-Diehl-2008
MT4	MT1E	0.023594758	Co-expression	Noble-Diehl-2008
MT4	MT1H	0.026304245	Co-expression	Noble-Diehl-2008
MT1X	MT1A	0.053587776	Co-expression	Noble-Diehl-2008
MT1X	MT1B	0.059370264	Co-expression	Noble-Diehl-2008
MT1X	MT1M	0.0505936	Co-expression	Noble-Diehl-2008
MT1X	MT1G	0.056908883	Co-expression	Noble-Diehl-2008
MT1X	MT1E	0.0438926	Co-expression	Noble-Diehl-2008
MT1X	MT1H	0.058808945	Co-expression	Noble-Diehl-2008
MT1X	MT1F	0.051111944	Co-expression	Noble-Diehl-2008
MT1X	MT4	0.02565249	Co-expression	Noble-Diehl-2008
MT2A	MT1A	0.053191002	Co-expression	Noble-Diehl-2008

MT2A	MT1B	0.060248733	Co-expression	Noble-Diehl-2008
MT2A	MT1M	0.047883872	Co-expression	Noble-Diehl-2008
MT2A	MT1G	0.056756314	Co-expression	Noble-Diehl-2008
MT2A	MT1E	0.04346904	Co-expression	Noble-Diehl-2008
MT2A	MT1H	0.058761757	Co-expression	Noble-Diehl-2008
MT2A	MT1F	0.048339766	Co-expression	Noble-Diehl-2008
MT2A	MT4	0.024605917	Co-expression	Noble-Diehl-2008
MT2A	MT1X	0.05959348	Co-expression	Noble-Diehl-2008
BBS2	MT1A	0.019398723	Co-expression	Noble-Diehl-2008
BBS2	MT1B	0.017454745	Co-expression	Noble-Diehl-2008
BBS2	MT1M	0.01768761	Co-expression	Noble-Diehl-2008
BBS2	MT1G	0.018586868	Co-expression	Noble-Diehl-2008
BBS2	MT1H	0.018527588	Co-expression	Noble-Diehl-2008
BBS2	MT1F	0.018282631	Co-expression	Noble-Diehl-2008
BBS2	MT1X	0.018482748	Co-expression	Noble-Diehl-2008
BBS2	MT2A	0.014477253	Co-expression	Noble-Diehl-2008
AAMDC	MT1A	0.017174695	Co-expression	Noble-Diehl-2008
AAMDC	MT1B	0.014906284	Co-expression	Noble-Diehl-2008
AAMDC	MT1M	0.016025364	Co-expression	Noble-Diehl-2008
AAMDC	MT1G	0.01611516	Co-expression	Noble-Diehl-2008
AAMDC	MT1H	0.015736826	Co-expression	Noble-Diehl-2008
AAMDC	MT1F	0.017035045	Co-expression	Noble-Diehl-2008
AAMDC	MT1X	0.015652105	Co-expression	Noble-Diehl-2008
AAMDC	MT2A	0.011273441	Co-expression	Noble-Diehl-2008
CD160	MT1B	0.02007477	Co-expression	Noble-Diehl-2008
CD160	MT1M	0.020379167	Co-expression	Noble-Diehl-2008
CD160	MT1G	0.019440776	Co-expression	Noble-Diehl-2008
CD160	MT1H	0.020016607	Co-expression	Noble-Diehl-2008
CD160	MT1X	0.020231128	Co-expression	Noble-Diehl-2008
CD160	MT2A	0.02138939	Co-expression	Noble-Diehl-2008
MARC2	MT1B	0.010311606	Co-expression	Noble-Diehl-2008
MARC2	MT1G	0.010660009	Co-expression	Noble-Diehl-2008
MARC2	MT1E	0.010389728	Co-expression	Noble-Diehl-2008
MARC2	MT1H	0.010579328	Co-expression	Noble-Diehl-2008
MARC2	MT1F	0.012290133	Co-expression	Noble-Diehl-2008
MARC2	MT4	0.011294243	Co-expression	Noble-Diehl-2008
MARC2	MT1X	0.01042546	Co-expression	Noble-Diehl-2008
TMEM51	MT1B	0.017014684	Co-expression	Noble-Diehl-2008
TMEM51	MT1E	0.018267347	Co-expression	Noble-Diehl-2008
TMEM51	MT1H	0.017890014	Co-expression	Noble-Diehl-2008
TMEM51	MT1X	0.016978435	Co-expression	Noble-Diehl-2008
TMEM51	MT2A	0.015092857	Co-expression	Noble-Diehl-2008
IYD	MT1M	0.010797457	Co-expression	Noble-Diehl-2008
IYD	MT1G	0.010902259	Co-expression	Noble-Diehl-2008

IYD	MT1E	0.011068492	Co-expression	Noble-Diehl-2008
IYD	MT1H	0.010463849	Co-expression	Noble-Diehl-2008
IYD	MT1F	0.0133128045	Co-expression	Noble-Diehl-2008
IYD	MT1X	0.01035977	Co-expression	Noble-Diehl-2008
IYD	MARC2	0.009611296	Co-expression	Noble-Diehl-2008
LGALS2	MT1B	0.015843278	Co-expression	Noble-Diehl-2008
LGALS2	MT1M	0.019010544	Co-expression	Noble-Diehl-2008
LGALS2	MT1G	0.015719041	Co-expression	Noble-Diehl-2008
LGALS2	MT1H	0.0154192	Co-expression	Noble-Diehl-2008
LGALS2	MT1F	0.020424537	Co-expression	Noble-Diehl-2008
LGALS2	MT1X	0.0153401075	Co-expression	Noble-Diehl-2008
LGALS2	IYD	0.0106974635	Co-expression	Noble-Diehl-2008
NEURL3	MT1G	0.017192682	Co-expression	Noble-Diehl-2008
NEURL3	MT1E	0.013783761	Co-expression	Noble-Diehl-2008
NEURL3	MT1H	0.016900292	Co-expression	Noble-Diehl-2008
NEURL3	MT1X	0.01712161	Co-expression	Noble-Diehl-2008
NEURL3	MT2A	0.018024618	Co-expression	Noble-Diehl-2008
C11orf52	MT1B	0.007734308	Co-expression	Noble-Diehl-2008
C11orf52	MT1G	0.008399046	Co-expression	Noble-Diehl-2008
C11orf52	MT1E	0.007911032	Co-expression	Noble-Diehl-2008
C11orf52	MT1H	0.007892154	Co-expression	Noble-Diehl-2008
C11orf52	MT1F	0.009630092	Co-expression	Noble-Diehl-2008
C11orf52	MT1X	0.0075621186	Co-expression	Noble-Diehl-2008
C11orf52	MARC2	0.007211665	Co-expression	Noble-Diehl-2008
C11orf52	TMEM51	0.0078984145	Co-expression	Noble-Diehl-2008
C11orf52	IYD	0.007004026	Co-expression	Noble-Diehl-2008
C11orf52	NEURL3	0.008031271	Co-expression	Noble-Diehl-2008
TMEM14C	MT1B	0.014533213	Co-expression	Noble-Diehl-2008
TMEM14C	MT1G	0.015461064	Co-expression	Noble-Diehl-2008
TMEM14C	MT1H	0.014917732	Co-expression	Noble-Diehl-2008
TMEM14C	MT1X	0.015447032	Co-expression	Noble-Diehl-2008
TMEM14C	MT2A	0.01333144	Co-expression	Noble-Diehl-2008
TMEM14C	AAMDC	0.013628003	Co-expression	Noble-Diehl-2008
TMEM14C	IYD	0.009999392	Co-expression	Noble-Diehl-2008

ZSWIM5	MT1M	0.012032025	Co-expression	Noble-Diehl-2008
ZSWIM5	MT1E	0.011097041	Co-expression	Noble-Diehl-2008
ZSWIM5	MT1H	0.0118575385	Co-expression	Noble-Diehl-2008
ZSWIM5	MT1F	0.014265556	Co-expression	Noble-Diehl-2008
ZSWIM5	MT1X	0.011457807	Co-expression	Noble-Diehl-2008
ZSWIM5	MARC2	0.009298708	Co-expression	Noble-Diehl-2008
ZSWIM5	IYD	0.009685226	Co-expression	Noble-Diehl-2008
ZSWIM5	C11orf52	0.007394623	Co-expression	Noble-Diehl-2008
SORBS3	MT1B	0.014684641	Co-expression	Noble-Diehl-2008
SORBS3	MT1G	0.016145784	Co-expression	Noble-Diehl-2008
SORBS3	MT1E	0.015635028	Co-expression	Noble-Diehl-2008
SORBS3	MT1H	0.016621163	Co-expression	Noble-Diehl-2008
SORBS3	MT1X	0.0155861955	Co-expression	Noble-Diehl-2008
TLR3	MT1B	0.011453399	Co-expression	Noble-Diehl-2008
TLR3	MT1H	0.011430469	Co-expression	Noble-Diehl-2008
TLR3	MT1X	0.011757491	Co-expression	Noble-Diehl-2008
TLR3	MT2A	0.011870267	Co-expression	Noble-Diehl-2008
TLR3	CD160	0.011174784	Co-expression	Noble-Diehl-2008
TLR3	IYD	0.008373018	Co-expression	Noble-Diehl-2008
MT1B	MT1A	0.056241788	Co-expression	Bild-Nevins-2006 B
MT1G	MT1A	0.040428516	Co-expression	Bild-Nevins-2006 B
MT1G	MT1B	0.04140584	Co-expression	Bild-Nevins-2006 B
MT1E	MT1A	0.055506386	Co-expression	Bild-Nevins-2006 B
MT1E	MT1B	0.060467377	Co-expression	Bild-Nevins-2006 B
MT1E	MT1G	0.034578282	Co-expression	Bild-Nevins-2006 B
MT1H	MT1A	0.055240706	Co-expression	Bild-Nevins-2006 B
MT1H	MT1B	0.05959477	Co-expression	Bild-Nevins-2006 B
MT1H	MT1G	0.050905343	Co-expression	Bild-Nevins-2006 B
MT1H	MT1E	0.053699452	Co-expression	Bild-Nevins-2006 B
MT1F	MT1A	0.055880807	Co-expression	Bild-Nevins-2006 B
MT1F	MT1B	0.058259383	Co-expression	Bild-Nevins-2006 B
MT1F	MT1G	0.04332734	Co-expression	Bild-Nevins-2006 B
MT1F	MT1E	0.0525224	Co-expression	Bild-Nevins-2006 B
MT1F	MT1H	0.059495762	Co-expression	Bild-Nevins-2006 B
MT1X	MT1A	0.036580216	Co-expression	Bild-Nevins-2006 B
MT1X	MT1B	0.028474007	Co-expression	Bild-Nevins-2006 B
MT1X	MT1G	0.014544422	Co-expression	Bild-Nevins-2006 B
MT1X	MT1E	0.03168932	Co-expression	Bild-Nevins-2006 B
MT1X	MT1H	0.027582372	Co-expression	Bild-Nevins-2006 B
MT1X	MT1F	0.029383702	Co-expression	Bild-Nevins-2006 B
MT3	MT1A	0.026096225	Co-expression	Bild-Nevins-2006 B
MT3	MT1B	0.030552814	Co-expression	Bild-Nevins-2006 B
MT3	MT1E	0.028922955	Co-expression	Bild-Nevins-2006 B

MT3	MT1H	0.02505486	Co-expression	Bild-Nevins-2006 B
MT3	MT1F	0.021442458	Co-expression	Bild-Nevins-2006 B
MT2A	MT1A	0.025590004	Co-expression	Bild-Nevins-2006 B
MT2A	MT1B	0.028114393	Co-expression	Bild-Nevins-2006 B
MT2A	MT1E	0.027606659	Co-expression	Bild-Nevins-2006 B
MT2A	MT1H	0.025839265	Co-expression	Bild-Nevins-2006 B
MT2A	MT1F	0.023897525	Co-expression	Bild-Nevins-2006 B
MT2A	MT1X	0.014686238	Co-expression	Bild-Nevins-2006 B
ASPA	MT1A	0.018377388	Co-expression	Bild-Nevins-2006 B
ASPA	MT1B	0.018643063	Co-expression	Bild-Nevins-2006 B
ASPA	MT1G	0.01927856	Co-expression	Bild-Nevins-2006 B
ASPA	MT1E	0.022199197	Co-expression	Bild-Nevins-2006 B
ASPA	MT1H	0.017346976	Co-expression	Bild-Nevins-2006 B
ASPA	MT2A	0.024357172	Co-expression	Bild-Nevins-2006 B
SYNM	MT1A	0.010726789	Co-expression	Bild-Nevins-2006 B
SYNM	MT1X	0.018379422	Co-expression	Bild-Nevins-2006 B
TNN	MT1A	0.017337395	Co-expression	Bild-Nevins-2006 B
TNN	MT1B	0.018987758	Co-expression	Bild-Nevins-2006 B
TNN	MT1E	0.020402748	Co-expression	Bild-Nevins-2006 B
TNN	MT1F	0.016379742	Co-expression	Bild-Nevins-2006 B
TNN	MT1X	0.016466983	Co-expression	Bild-Nevins-2006 B
TLR3	MT1B	0.02566196	Co-expression	Bild-Nevins-2006 B
TLR3	MT1H	0.02023964	Co-expression	Bild-Nevins-2006 B
TLR3	CD160	0.015939916	Co-expression	Bild-Nevins-2006 B
MT1B	MT1A	0.036396068	Co-expression	Burington-Shaughnessy-2008
MT1G	MT1A	0.050568495	Co-expression	Burington-Shaughnessy-2008
MT1G	MT1B	0.030821707	Co-expression	Burington-Shaughnessy-2008
MT1E	MT1A	0.031996038	Co-expression	Burington-Shaughnessy-2008
MT1E	MT1B	0.028395893	Co-expression	Burington-Shaughnessy-2008
MT1E	MT1G	0.030432383	Co-expression	Burington-Shaughnessy-2008
MT1H	MT1A	0.04788622	Co-expression	Burington-Shaughnessy-2008
MT1H	MT1B	0.03773679	Co-expression	Burington-Shaughnessy-2008
MT1H	MT1G	0.04877982	Co-expression	Burington-Shaughnessy-2008
MT1H	MT1E	0.034547456	Co-expression	Burington-Shaughnessy-2008

MT1F MT1A 0.05508056 Co-expression
Burington-Shaughnessy-2008
MT1F MT1B 0.03818799 Co-expression
Burington-Shaughnessy-2008
MT1F MT1G 0.04574338 Co-expression
Burington-Shaughnessy-2008
MT1F MT1E 0.034765914 Co-expression
Burington-Shaughnessy-2008
MT1F MT1H 0.049423125 Co-expression
Burington-Shaughnessy-2008
MT1X MT1A 0.046169303 Co-expression
Burington-Shaughnessy-2008
MT1X MT1B 0.033873923 Co-expression
Burington-Shaughnessy-2008
MT1X MT1G 0.042088587 Co-expression
Burington-Shaughnessy-2008
MT1X MT1E 0.033666994 Co-expression
Burington-Shaughnessy-2008
MT1X MT1H 0.04723908 Co-expression
Burington-Shaughnessy-2008
MT1X MT1F 0.04897226 Co-expression
Burington-Shaughnessy-2008
MT3 MT1A 0.02647599 Co-expression
Burington-Shaughnessy-2008
MT3 MT1B 0.026231822 Co-expression
Burington-Shaughnessy-2008
MT3 MT1G 0.024561139 Co-expression
Burington-Shaughnessy-2008
MT3 MT1E 0.021731801 Co-expression
Burington-Shaughnessy-2008
MT3 MT1H 0.027681671 Co-expression
Burington-Shaughnessy-2008
MT3 MT1F 0.024921983 Co-expression
Burington-Shaughnessy-2008
MT3 MT1X 0.023390753 Co-expression
Burington-Shaughnessy-2008
MT2A MT1B 0.00924687 Co-expression
Burington-Shaughnessy-2008
MT2A MT1E 0.008809023 Co-expression
Burington-Shaughnessy-2008
MT2A MT1H 0.008513502 Co-expression
Burington-Shaughnessy-2008
MT2A MT1F 0.0077223843 Co-expression
Burington-Shaughnessy-2008

MT2A	MT1X	0.008533018	Co-expression Burington-Shaughnessy-2008
ASPA	MT2A	0.011980948	Co-expression Burington-Shaughnessy-2008
PTGDR	MT1B	0.02335423	Co-expression Burington-Shaughnessy-2008
PTGDR	MT1G	0.031006133	Co-expression Burington-Shaughnessy-2008
PTGDR	MT1E	0.021492634	Co-expression Burington-Shaughnessy-2008
PTGDR	MT1H	0.029531814	Co-expression Burington-Shaughnessy-2008
PTGDR	MT1F	0.027270975	Co-expression Burington-Shaughnessy-2008
PTGDR	MT1X	0.01799441	Co-expression Burington-Shaughnessy-2008
PTGDR	MT2A	0.008630991	Co-expression Burington-Shaughnessy-2008
SPP1	MT1B	0.015549186	Co-expression Burington-Shaughnessy-2008
SPP1	MT1G	0.028138248	Co-expression Burington-Shaughnessy-2008
SPP1	MT1E	0.014938028	Co-expression Burington-Shaughnessy-2008
SPP1	MT1H	0.023792041	Co-expression Burington-Shaughnessy-2008
SPP1	MT1F	0.02498577	Co-expression Burington-Shaughnessy-2008
SYNM	MT1A	0.020261927	Co-expression Burington-Shaughnessy-2008
SYNM	MT1G	0.02512907	Co-expression Burington-Shaughnessy-2008
SYNM	MT1E	0.015088857	Co-expression Burington-Shaughnessy-2008
SYNM	MT1H	0.019958287	Co-expression Burington-Shaughnessy-2008
SYNM	MT1F	0.019820943	Co-expression Burington-Shaughnessy-2008
SYNM	MT1X	0.026271354	Co-expression Burington-Shaughnessy-2008
ACPP	MT1G	0.031436183	Co-expression Burington-Shaughnessy-2008
ACPP	MT1E	0.019822188	Co-expression Burington-Shaughnessy-2008

ACPP	MT1H	0.02896131	Co-expression	Burington-Shaughnessy-2008
ACPP	MT1F	0.028248925	Co-expression	Burington-Shaughnessy-2008
ACPP	MT3	0.022997642	Co-expression	Burington-Shaughnessy-2008
ACPP	PTGDR	0.025313215	Co-expression	Burington-Shaughnessy-2008
MT1B	MT1A	0.0403171	Co-expression	Innocenti-Brown-2011
MT1M	MT1A	0.028606279	Co-expression	Innocenti-Brown-2011
MT1M	MT1B	0.023235887	Co-expression	Innocenti-Brown-2011
MT1G	MT1A	0.044731762	Co-expression	Innocenti-Brown-2011
MT1G	MT1B	0.04254193	Co-expression	Innocenti-Brown-2011
MT1G	MT1M	0.02691225	Co-expression	Innocenti-Brown-2011
MT1E	MT1A	0.037636552	Co-expression	Innocenti-Brown-2011
MT1E	MT1B	0.034266308	Co-expression	Innocenti-Brown-2011
MT1E	MT1M	0.025075862	Co-expression	Innocenti-Brown-2011
MT1E	MT1G	0.038911305	Co-expression	Innocenti-Brown-2011
MT1H	MT1A	0.041996278	Co-expression	Innocenti-Brown-2011
MT1H	MT1B	0.03873715	Co-expression	Innocenti-Brown-2011
MT1H	MT1M	0.025751697	Co-expression	Innocenti-Brown-2011
MT1H	MT1G	0.043205865	Co-expression	Innocenti-Brown-2011
MT1H	MT1E	0.036010228	Co-expression	Innocenti-Brown-2011
MT1F	MT1A	0.029022299	Co-expression	Innocenti-Brown-2011
MT1F	MT1B	0.025331948	Co-expression	Innocenti-Brown-2011
MT1F	MT1M	0.020878008	Co-expression	Innocenti-Brown-2011
MT1F	MT1G	0.037532207	Co-expression	Innocenti-Brown-2011
MT1F	MT1E	0.0320617	Co-expression	Innocenti-Brown-2011
MT1F	MT1H	0.026256423	Co-expression	Innocenti-Brown-2011
MT1X	MT1A	0.03948276	Co-expression	Innocenti-Brown-2011
MT1X	MT1B	0.03769043	Co-expression	Innocenti-Brown-2011
MT1X	MT1M	0.022977468	Co-expression	Innocenti-Brown-2011
MT1X	MT1G	0.04172955	Co-expression	Innocenti-Brown-2011
MT1X	MT1E	0.035192166	Co-expression	Innocenti-Brown-2011
MT1X	MT1H	0.038517136	Co-expression	Innocenti-Brown-2011
MT1X	MT1F	0.025273787	Co-expression	Innocenti-Brown-2011
MT2A	MT1A	0.0361544	Co-expression	Innocenti-Brown-2011
MT2A	MT1B	0.037574362	Co-expression	Innocenti-Brown-2011
MT2A	MT1M	0.02038164	Co-expression	Innocenti-Brown-2011
MT2A	MT1G	0.038609505	Co-expression	Innocenti-Brown-2011
MT2A	MT1E	0.03394773	Co-expression	Innocenti-Brown-2011
MT2A	MT1H	0.033855703	Co-expression	Innocenti-Brown-2011
MT2A	MT1F	0.024946308	Co-expression	Innocenti-Brown-2011
MT2A	MT1X	0.035128742	Co-expression	Innocenti-Brown-2011

MARC2	MT1F	0.014885814	Co-expression	Innocenti-Brown-2011
TMEM51	BBS2	0.004407377	Co-expression	Innocenti-Brown-2011
PTGDR	CD160	0.017097695	Co-expression	Innocenti-Brown-2011
MT1B	MT1A	0.03034516	Co-expression	Mallon-McKay-2013
MT1M	MT1A	0.024813067	Co-expression	Mallon-McKay-2013
MT1M	MT1B	0.02735597	Co-expression	Mallon-McKay-2013
MT1G	MT1A	0.02734936	Co-expression	Mallon-McKay-2013
MT1G	MT1B	0.029725213	Co-expression	Mallon-McKay-2013
MT1G	MT1M	0.025358796	Co-expression	Mallon-McKay-2013
MT1E	MT1A	0.024529362	Co-expression	Mallon-McKay-2013
MT1E	MT1B	0.027382359	Co-expression	Mallon-McKay-2013
MT1E	MT1M	0.025718847	Co-expression	Mallon-McKay-2013
MT1E	MT1G	0.025307352	Co-expression	Mallon-McKay-2013
MT1H	MT1A	0.028544432	Co-expression	Mallon-McKay-2013
MT1H	MT1B	0.030594213	Co-expression	Mallon-McKay-2013
MT1H	MT1M	0.025327964	Co-expression	Mallon-McKay-2013
MT1H	MT1G	0.027623925	Co-expression	Mallon-McKay-2013
MT1H	MT1E	0.024715696	Co-expression	Mallon-McKay-2013
MT1F	MT1A	0.019200174	Co-expression	Mallon-McKay-2013
MT1F	MT1B	0.020852301	Co-expression	Mallon-McKay-2013
MT1F	MT1M	0.018963544	Co-expression	Mallon-McKay-2013
MT1F	MT1G	0.021063197	Co-expression	Mallon-McKay-2013
MT1F	MT1E	0.018985951	Co-expression	Mallon-McKay-2013
MT1F	MT1H	0.019012323	Co-expression	Mallon-McKay-2013
MT1X	MT1A	0.030218374	Co-expression	Mallon-McKay-2013
MT1X	MT1B	0.032149725	Co-expression	Mallon-McKay-2013
MT1X	MT1M	0.025775233	Co-expression	Mallon-McKay-2013
MT1X	MT1G	0.028825445	Co-expression	Mallon-McKay-2013
MT1X	MT1E	0.026289228	Co-expression	Mallon-McKay-2013
MT1X	MT1H	0.030587073	Co-expression	Mallon-McKay-2013
MT1X	MT1F	0.01927198	Co-expression	Mallon-McKay-2013
MT2A	MT1A	0.021399569	Co-expression	Mallon-McKay-2013
MT2A	MT1B	0.023521952	Co-expression	Mallon-McKay-2013
MT2A	MT1G	0.018771064	Co-expression	Mallon-McKay-2013
MT2A	MT1H	0.021466902	Co-expression	Mallon-McKay-2013
MT2A	MT1X	0.023265455	Co-expression	Mallon-McKay-2013
MARC2	MT1B	0.016491363	Co-expression	Mallon-McKay-2013
MARC2	MT1M	0.016025431	Co-expression	Mallon-McKay-2013
MARC2	MT1G	0.016362872	Co-expression	Mallon-McKay-2013
MARC2	MT1E	0.01277978	Co-expression	Mallon-McKay-2013
MARC2	MT1H	0.015224094	Co-expression	Mallon-McKay-2013
MARC2	MT1F	0.014206209	Co-expression	Mallon-McKay-2013
MARC2	MT1X	0.014825863	Co-expression	Mallon-McKay-2013
IYD	MT1F	0.014980373	Co-expression	Mallon-McKay-2013

C11orf52	MT1F	0.012311523	Co-expression	Mallon-McKay-2013
TMEM14C	AAMDC	0.014353856	Co-expression	Mallon-McKay-2013
MT1B	MT1A	0.016802212	Co-expression	Rieger-Chu-2004
MT1G	MT1A	0.017683422	Co-expression	Rieger-Chu-2004
MT1G	MT1B	0.016321868	Co-expression	Rieger-Chu-2004
MT1E	MT1A	0.0248974	Co-expression	Rieger-Chu-2004
MT1E	MT1B	0.021092933	Co-expression	Rieger-Chu-2004
MT1E	MT1G	0.01785389	Co-expression	Rieger-Chu-2004
MT1H	MT1A	0.020233829	Co-expression	Rieger-Chu-2004
MT1H	MT1B	0.017067445	Co-expression	Rieger-Chu-2004
MT1H	MT1G	0.017085792	Co-expression	Rieger-Chu-2004
MT1H	MT1E	0.023163449	Co-expression	Rieger-Chu-2004
MT1F	MT1A	0.02772654	Co-expression	Rieger-Chu-2004
MT1F	MT1B	0.017795973	Co-expression	Rieger-Chu-2004
MT1F	MT1G	0.017098246	Co-expression	Rieger-Chu-2004
MT1F	MT1E	0.026268957	Co-expression	Rieger-Chu-2004
MT1F	MT1H	0.021819677	Co-expression	Rieger-Chu-2004
MT1X	MT1A	0.020436224	Co-expression	Rieger-Chu-2004
MT1X	MT1B	0.014104216	Co-expression	Rieger-Chu-2004
MT1X	MT1E	0.023152381	Co-expression	Rieger-Chu-2004
MT1X	MT1H	0.017269947	Co-expression	Rieger-Chu-2004
MT1X	MT1F	0.017475067	Co-expression	Rieger-Chu-2004
MT3	MT1A	0.023454573	Co-expression	Rieger-Chu-2004
MT3	MT1B	0.015391358	Co-expression	Rieger-Chu-2004
MT3	MT1H	0.013468642	Co-expression	Rieger-Chu-2004
MT3	MT1F	0.025220785	Co-expression	Rieger-Chu-2004
MT2A	MT1A	0.020572137	Co-expression	Rieger-Chu-2004
MT2A	MT1B	0.019739201	Co-expression	Rieger-Chu-2004
MT2A	MT1G	0.018344646	Co-expression	Rieger-Chu-2004
MT2A	MT1E	0.021812404	Co-expression	Rieger-Chu-2004
MT2A	MT1H	0.02132555	Co-expression	Rieger-Chu-2004
MT2A	MT1F	0.021975115	Co-expression	Rieger-Chu-2004
SPP1	MT1B	0.012277597	Co-expression	Rieger-Chu-2004
TNN	MT1A	0.01638659	Co-expression	Rieger-Chu-2004
TNN	MT1B	0.011036408	Co-expression	Rieger-Chu-2004
TNN	MT1E	0.013432498	Co-expression	Rieger-Chu-2004
TNN	MT1H	0.01454941	Co-expression	Rieger-Chu-2004
TNN	MT1F	0.014570819	Co-expression	Rieger-Chu-2004
TNN	MT2A	0.013961939	Co-expression	Rieger-Chu-2004
TNN	SPP1	0.018318765	Co-expression	Rieger-Chu-2004
MT1E	MT1G	0.047637615	Co-expression	Bahr-Bowler-2013
MT1F	MT1G	0.047751512	Co-expression	Bahr-Bowler-2013
MT1F	MT1E	0.049832836	Co-expression	Bahr-Bowler-2013

MT1X	MT1G	0.04849785	Co-expression	Bahr-Bowler-2013
MT1X	MT1E	0.049828872	Co-expression	Bahr-Bowler-2013
MT1X	MT1F	0.05134176	Co-expression	Bahr-Bowler-2013
MT2A	MT1G	0.048610903	Co-expression	Bahr-Bowler-2013
MT2A	MT1E	0.048932143	Co-expression	Bahr-Bowler-2013
MT2A	MT1F	0.04706711	Co-expression	Bahr-Bowler-2013
MT2A	MT1X	0.049489282	Co-expression	Bahr-Bowler-2013
PTGDR	CD160	0.012078402	Co-expression	Bahr-Bowler-2013
MT1B	MT1A	0.017300155	Co-expression	Wu-Garvey-2007
MT1E	MT1A	0.020082666	Co-expression	Wu-Garvey-2007
MT1E	MT1B	0.020257832	Co-expression	Wu-Garvey-2007
MT1H	MT1A	0.021279566	Co-expression	Wu-Garvey-2007
MT1H	MT1B	0.018874256	Co-expression	Wu-Garvey-2007
MT1H	MT1E	0.021950554	Co-expression	Wu-Garvey-2007
MT1F	MT1A	0.0199854	Co-expression	Wu-Garvey-2007
MT1F	MT1B	0.018221732	Co-expression	Wu-Garvey-2007
MT1F	MT1E	0.021059778	Co-expression	Wu-Garvey-2007
MT1F	MT1H	0.02243226	Co-expression	Wu-Garvey-2007
MT1X	MT1A	0.020700807	Co-expression	Wu-Garvey-2007
MT1X	MT1B	0.019337362	Co-expression	Wu-Garvey-2007
MT1X	MT1E	0.022014707	Co-expression	Wu-Garvey-2007
MT1X	MT1H	0.023250189	Co-expression	Wu-Garvey-2007
MT1X	MT1F	0.021345655	Co-expression	Wu-Garvey-2007
MT1E	MT1B	0.013343066	Co-expression	Wang-Maris-2006
MT1H	MT1B	0.01663165	Co-expression	Wang-Maris-2006
MT1H	MT1E	0.014874805	Co-expression	Wang-Maris-2006
MT1F	MT1A	0.008840001	Co-expression	Wang-Maris-2006
MT1F	MT1B	0.016757714	Co-expression	Wang-Maris-2006
MT1F	MT1E	0.014779578	Co-expression	Wang-Maris-2006
MT1F	MT1H	0.01970675	Co-expression	Wang-Maris-2006
MT1X	MT1B	0.011906932	Co-expression	Wang-Maris-2006
MT1X	MT1E	0.009231975	Co-expression	Wang-Maris-2006
MT1X	MT1H	0.016113248	Co-expression	Wang-Maris-2006
MT1X	MT1F	0.016340926	Co-expression	Wang-Maris-2006
MT3	MT1A	0.012993147	Co-expression	Wang-Maris-2006
MT2A	MT1B	0.0142735895	Co-expression	Wang-Maris-2006
MT2A	MT1E	0.013293312	Co-expression	Wang-Maris-2006
MT2A	MT1H	0.016232643	Co-expression	Wang-Maris-2006
MT2A	MT1F	0.016650379	Co-expression	Wang-Maris-2006
ACPP	MT3	0.011482083	Co-expression	Wang-Maris-2006
MT1E	MT1G	0.018407654	Co-expression	Ramaswamy-Golub-2001
MT1H	MT1G	0.01773741	Co-expression	Ramaswamy-Golub-2001
MT1H	MT1E	0.019687425	Co-expression	Ramaswamy-Golub-2001
MT1F	MT1G	0.015495117	Co-expression	Ramaswamy-Golub-2001

MT1F	MT1E	0.018362675	Co-expression	Ramaswamy-Golub-2001
MT1X	MT1G	0.013210557	Co-expression	Ramaswamy-Golub-2001
MT1X	MT1E	0.019136544	Co-expression	Ramaswamy-Golub-2001
MT1X	MT1H	0.01195355	Co-expression	Ramaswamy-Golub-2001
MT1X	MT1F	0.016499944	Co-expression	Ramaswamy-Golub-2001
MT2A	MT1G	0.017624835	Co-expression	Ramaswamy-Golub-2001
MT2A	MT1E	0.022562286	Co-expression	Ramaswamy-Golub-2001
MT2A	MT1H	0.024701927	Co-expression	Ramaswamy-Golub-2001
ACPP	SYNM	0.0077964915	Co-expression	Ramaswamy-Golub-2001
MT1E	MT1M	0.011397265	Co-expression	Smirnov-Cheung-2009
MT1E	MT1G	0.015733035	Co-expression	Smirnov-Cheung-2009
MT1H	MT1M	0.011773016	Co-expression	Smirnov-Cheung-2009
MT1H	MT1G	0.016022312	Co-expression	Smirnov-Cheung-2009
MT1H	MT1E	0.010073123	Co-expression	Smirnov-Cheung-2009
MT1F	MT1M	0.024012852	Co-expression	Smirnov-Cheung-2009
MT1F	MT1G	0.028578337	Co-expression	Smirnov-Cheung-2009
MT1F	MT1E	0.017846905	Co-expression	Smirnov-Cheung-2009
MT1F	MT1H	0.017690456	Co-expression	Smirnov-Cheung-2009
MT1X	MT1M	0.009755845	Co-expression	Smirnov-Cheung-2009
MT1X	MT1G	0.013193002	Co-expression	Smirnov-Cheung-2009
MT1X	MT1E	0.008322022	Co-expression	Smirnov-Cheung-2009
MT1X	MT1H	0.008860818	Co-expression	Smirnov-Cheung-2009
MT1X	MT1F	0.014856434	Co-expression	Smirnov-Cheung-2009
MT2A	MT1M	0.009000205	Co-expression	Smirnov-Cheung-2009
MT2A	MT1G	0.012364408	Co-expression	Smirnov-Cheung-2009
MT2A	MT1E	0.0070261694	Co-expression	Smirnov-Cheung-2009
MT2A	MT1H	0.0073463796	Co-expression	Smirnov-Cheung-2009
MT2A	MT1F	0.013033768	Co-expression	Smirnov-Cheung-2009
MT2A	MT1X	0.006337821	Co-expression	Smirnov-Cheung-2009
MT1HL1	MT1M	0.011029311	Co-expression	Smirnov-Cheung-2009
MT1HL1	MT1G	0.013150788	Co-expression	Smirnov-Cheung-2009
MT1HL1	MT1E	0.0083202645	Co-expression	Smirnov-Cheung-2009
MT1HL1	MT1H	0.009030377	Co-expression	Smirnov-Cheung-2009
MT1HL1	MT1F	0.0149915395	Co-expression	Smirnov-Cheung-2009
MT1HL1	MT1X	0.007683658	Co-expression	Smirnov-Cheung-2009
MT1HL1	MT2A	0.0063241324	Co-expression	Smirnov-Cheung-2009
SYNM	MT1M	0.015044341	Co-expression	Smirnov-Cheung-2009
SYNM	MT1X	0.0060453815	Co-expression	Smirnov-Cheung-2009
SYNM	MT2A	0.005745621	Co-expression	Smirnov-Cheung-2009
SYNM	MT1HL1	0.006698264	Co-expression	Smirnov-Cheung-2009
MT1H	MT1G	0.03965586	Co-expression	Perou-Botstein-2000
MT1X	MT1G	0.02192262	Co-expression	Perou-Botstein-2000
MT1X	MT1H	0.01699814	Co-expression	Perou-Botstein-2000
MT1E	MT1M	0.020685583	Co-expression	Wang-Cheung-2015

MT1E	MT1G	0.029589344	Co-expression	Wang-Cheung-2015
MT1H	MT1G	0.021374265	Co-expression	Wang-Cheung-2015
MT1H	MT1E	0.024106892	Co-expression	Wang-Cheung-2015
MT1F	MT1M	0.026605982	Co-expression	Wang-Cheung-2015
MT1F	MT1G	0.021847313	Co-expression	Wang-Cheung-2015
MT1F	MT1E	0.03593919	Co-expression	Wang-Cheung-2015
MT1F	MT1H	0.017233187	Co-expression	Wang-Cheung-2015
MT1X	MT1M	0.021664599	Co-expression	Wang-Cheung-2015
MT1X	MT1G	0.033924114	Co-expression	Wang-Cheung-2015
MT1X	MT1E	0.038827438	Co-expression	Wang-Cheung-2015
MT1X	MT1H	0.026530085	Co-expression	Wang-Cheung-2015
MT1X	MT1F	0.03202094	Co-expression	Wang-Cheung-2015
MT2A	MT1G	0.018752046	Co-expression	Wang-Cheung-2015
MT2A	MT1E	0.023072122	Co-expression	Wang-Cheung-2015
MT2A	MT1H	0.017567841	Co-expression	Wang-Cheung-2015
MT2A	MT1X	0.021297805	Co-expression	Wang-Cheung-2015
MT1HL1	MT1M	0.013571696	Co-expression	Wang-Cheung-2015
MT1HL1	MT1G	0.021641666	Co-expression	Wang-Cheung-2015
MT1HL1	MT1E	0.024674367	Co-expression	Wang-Cheung-2015
MT1HL1	MT1H	0.020194355	Co-expression	Wang-Cheung-2015
MT1HL1	MT1F	0.023817746	Co-expression	Wang-Cheung-2015
MT1HL1	MT1X	0.033118032	Co-expression	Wang-Cheung-2015
MT1HL1	MT2A	0.015921522	Co-expression	Wang-Cheung-2015
TMEM51	MARC2	0.011158744	Co-expression	Wang-Cheung-2015
TLR3	MARC2	0.009057841	Co-expression	Wang-Cheung-2015
MT1G	MT1M	0.012954659	Co-expression	Dobbin-Giordano-2005
MT1E	MT1M	0.009593395	Co-expression	Dobbin-Giordano-2005
MT1E	MT1G	0.017209582	Co-expression	Dobbin-Giordano-2005
MT1H	MT1M	0.0104819415	Co-expression	Dobbin-Giordano-2005
MT1H	MT1G	0.018528355	Co-expression	Dobbin-Giordano-2005
MT1H	MT1E	0.022883251	Co-expression	Dobbin-Giordano-2005
MT1F	MT1M	0.009747206	Co-expression	Dobbin-Giordano-2005
MT1F	MT1G	0.01677403	Co-expression	Dobbin-Giordano-2005
MT1F	MT1E	0.015167352	Co-expression	Dobbin-Giordano-2005
MT1F	MT1H	0.015565681	Co-expression	Dobbin-Giordano-2005
MT1X	MT1M	0.01032614	Co-expression	Dobbin-Giordano-2005
MT1X	MT1G	0.018575994	Co-expression	Dobbin-Giordano-2005
MT1X	MT1E	0.022142341	Co-expression	Dobbin-Giordano-2005
MT1X	MT1H	0.021824732	Co-expression	Dobbin-Giordano-2005
MT1X	MT1F	0.015656749	Co-expression	Dobbin-Giordano-2005
MT2A	MT1M	0.009456251	Co-expression	Dobbin-Giordano-2005
MT2A	MT1G	0.01794447	Co-expression	Dobbin-Giordano-2005
MT2A	MT1E	0.02468447	Co-expression	Dobbin-Giordano-2005
MT2A	MT1H	0.02371543	Co-expression	Dobbin-Giordano-2005

MT2A	MT1F	0.015640887	Co-expression	Dobbin-Giordano-2005
MT2A	MT1X	0.024217762	Co-expression	Dobbin-Giordano-2005
MT1HL1	MT1M	0.011022267	Co-expression	Dobbin-Giordano-2005
MT1HL1	MT1G	0.01982003	Co-expression	Dobbin-Giordano-2005
MT1HL1	MT1E	0.023410695	Co-expression	Dobbin-Giordano-2005
MT1HL1	MT1H	0.02360374	Co-expression	Dobbin-Giordano-2005
MT1HL1	MT1F	0.016279355	Co-expression	Dobbin-Giordano-2005
MT1HL1	MT1X	0.022088528	Co-expression	Dobbin-Giordano-2005
MT1HL1	MT2A	0.024438627	Co-expression	Dobbin-Giordano-2005
MT1H	MT1G	0.008816806	Co-expression	Chen-Brown-2002
MT1X	MT1G	0.0068854634	Co-expression	Chen-Brown-2002
MT1X	MT1H	0.0067705624	Co-expression	Chen-Brown-2002
TLR3	ASPA	0.02062715	Co-expression	Chen-Brown-2002
MT1H	MT1G	0.010814037	Co-localization	Johnson-Shoemaker-2003
MT3	MT1G	0.015239366	Co-localization	Johnson-Shoemaker-2003
MT3	MT1H	0.016063996	Co-localization	Johnson-Shoemaker-2003
SPP1	ASPA	0.00798056	Co-localization	Johnson-Shoemaker-2003
MT1G	MT1M	0.21846053	Predicted	Wu-Stein-2010
MT1E	MT1G	0.18305619	Predicted	Wu-Stein-2010
MT1H	MT1M	0.21846053	Predicted	Wu-Stein-2010
MT1H	MT1G	0.15586922	Predicted	Wu-Stein-2010
MT1H	MT1E	0.18305619	Predicted	Wu-Stein-2010
MT1F	MT1M	0.21846053	Predicted	Wu-Stein-2010
MT1F	MT1G	0.15586922	Predicted	Wu-Stein-2010
MT1F	MT1E	0.18305619	Predicted	Wu-Stein-2010
MT1F	MT1H	0.15586922	Predicted	Wu-Stein-2010
MT1X	MT1M	0.21846053	Predicted	Wu-Stein-2010
MT1X	MT1G	0.15586922	Predicted	Wu-Stein-2010
MT1X	MT1E	0.18305619	Predicted	Wu-Stein-2010
MT1X	MT1H	0.15586922	Predicted	Wu-Stein-2010
MT1X	MT1F	0.15586922	Predicted	Wu-Stein-2010
MT2A	MT1G	0.18305619	Predicted	Wu-Stein-2010
MT2A	MT1E	0.21498518	Predicted	Wu-Stein-2010
MT2A	MT1H	0.18305619	Predicted	Wu-Stein-2010
MT2A	MT1F	0.18305619	Predicted	Wu-Stein-2010
MT2A	MT1X	0.18305619	Predicted	Wu-Stein-2010
MT1B	MT1A	0.09516845	Shared protein domains	INTERPRO
MT1M	MT1A	0.09516845	Shared protein domains	INTERPRO
MT1M	MT1B	0.09516845	Shared protein domains	INTERPRO
MT1G	MT1A	0.09516845	Shared protein domains	INTERPRO
MT1G	MT1B	0.09516845	Shared protein domains	INTERPRO
MT1G	MT1M	0.09516845	Shared protein domains	INTERPRO
MT1E	MT1A	0.09516845	Shared protein domains	INTERPRO
MT1E	MT1B	0.09516845	Shared protein domains	INTERPRO

MT1E	MT1M	0.09516845	Shared protein domains INTERPRO
MT1E	MT1G	0.09516845	Shared protein domains INTERPRO
MT1H	MT1A	0.09516845	Shared protein domains INTERPRO
MT1H	MT1B	0.09516845	Shared protein domains INTERPRO
MT1H	MT1M	0.09516845	Shared protein domains INTERPRO
MT1H	MT1G	0.09516845	Shared protein domains INTERPRO
MT1H	MT1E	0.09516845	Shared protein domains INTERPRO
MT1F	MT1A	0.09516845	Shared protein domains INTERPRO
MT1F	MT1B	0.09516845	Shared protein domains INTERPRO
MT1F	MT1M	0.09516845	Shared protein domains INTERPRO
MT1F	MT1G	0.09516845	Shared protein domains INTERPRO
MT1F	MT1E	0.09516845	Shared protein domains INTERPRO
MT1F	MT1H	0.09516845	Shared protein domains INTERPRO
MT4	MT1A	0.085647054	Shared protein domains INTERPRO
MT4	MT1B	0.085647054	Shared protein domains INTERPRO
MT4	MT1M	0.085647054	Shared protein domains INTERPRO
MT4	MT1G	0.085647054	Shared protein domains INTERPRO
MT4	MT1E	0.085647054	Shared protein domains INTERPRO
MT4	MT1H	0.085647054	Shared protein domains INTERPRO
MT4	MT1F	0.085647054	Shared protein domains INTERPRO
MT1X	MT1A	0.09516845	Shared protein domains INTERPRO
MT1X	MT1B	0.09516845	Shared protein domains INTERPRO
MT1X	MT1M	0.09516845	Shared protein domains INTERPRO
MT1X	MT1G	0.09516845	Shared protein domains INTERPRO
MT1X	MT1E	0.09516845	Shared protein domains INTERPRO
MT1X	MT1H	0.09516845	Shared protein domains INTERPRO
MT1X	MT1F	0.09516845	Shared protein domains INTERPRO
MT1X	MT4	0.085647054	Shared protein domains INTERPRO
MT3	MT1A	0.09516845	Shared protein domains INTERPRO
MT3	MT1B	0.09516845	Shared protein domains INTERPRO
MT3	MT1M	0.09516845	Shared protein domains INTERPRO
MT3	MT1G	0.09516845	Shared protein domains INTERPRO
MT3	MT1E	0.09516845	Shared protein domains INTERPRO
MT3	MT1H	0.09516845	Shared protein domains INTERPRO
MT3	MT1F	0.09516845	Shared protein domains INTERPRO
MT3	MT4	0.085647054	Shared protein domains INTERPRO
MT3	MT1X	0.09516845	Shared protein domains INTERPRO
MT2A	MT1A	0.09516845	Shared protein domains INTERPRO
MT2A	MT1B	0.09516845	Shared protein domains INTERPRO
MT2A	MT1M	0.09516845	Shared protein domains INTERPRO
MT2A	MT1G	0.09516845	Shared protein domains INTERPRO
MT2A	MT1E	0.09516845	Shared protein domains INTERPRO
MT2A	MT1H	0.09516845	Shared protein domains INTERPRO
MT2A	MT1F	0.09516845	Shared protein domains INTERPRO

MT2A	MT4	0.085647054	Shared protein domains INTERPRO
MT2A	MT1X	0.09516845	Shared protein domains INTERPRO
MT2A	MT3	0.09516845	Shared protein domains INTERPRO
MT1HL1	MT1A	0.05855918	Shared protein domains INTERPRO
MT1HL1	MT1B	0.05855918	Shared protein domains INTERPRO
MT1HL1	MT1M	0.05855918	Shared protein domains INTERPRO
MT1HL1	MT1G	0.05855918	Shared protein domains INTERPRO
MT1HL1	MT1E	0.05855918	Shared protein domains INTERPRO
MT1HL1	MT1H	0.05855918	Shared protein domains INTERPRO
MT1HL1	MT1F	0.05855918	Shared protein domains INTERPRO
MT1HL1	MT4	0.071133636	Shared protein domains INTERPRO
MT1HL1	MT1X	0.05855918	Shared protein domains INTERPRO
MT1HL1	MT3	0.05855918	Shared protein domains INTERPRO
MT1HL1	MT2A	0.05855918	Shared protein domains INTERPRO
MT1B	MT1A	0.09090909	Shared protein domains PFAM
MT1M	MT1A	0.09090909	Shared protein domains PFAM
MT1M	MT1B	0.09090909	Shared protein domains PFAM
MT1G	MT1A	0.09090909	Shared protein domains PFAM
MT1G	MT1B	0.09090909	Shared protein domains PFAM
MT1G	MT1M	0.09090909	Shared protein domains PFAM
MT1E	MT1A	0.09090909	Shared protein domains PFAM
MT1E	MT1B	0.09090909	Shared protein domains PFAM
MT1E	MT1M	0.09090909	Shared protein domains PFAM
MT1E	MT1G	0.09090909	Shared protein domains PFAM
MT1H	MT1A	0.09090909	Shared protein domains PFAM
MT1H	MT1B	0.09090909	Shared protein domains PFAM
MT1H	MT1M	0.09090909	Shared protein domains PFAM
MT1H	MT1G	0.09090909	Shared protein domains PFAM
MT1H	MT1E	0.09090909	Shared protein domains PFAM
MT1F	MT1A	0.09090909	Shared protein domains PFAM
MT1F	MT1B	0.09090909	Shared protein domains PFAM
MT1F	MT1M	0.09090909	Shared protein domains PFAM
MT1F	MT1G	0.09090909	Shared protein domains PFAM
MT1F	MT1E	0.09090909	Shared protein domains PFAM
MT1F	MT1H	0.09090909	Shared protein domains PFAM
MT4	MT1A	0.09090909	Shared protein domains PFAM
MT4	MT1B	0.09090909	Shared protein domains PFAM
MT4	MT1M	0.09090909	Shared protein domains PFAM
MT4	MT1G	0.09090909	Shared protein domains PFAM
MT4	MT1E	0.09090909	Shared protein domains PFAM
MT4	MT1H	0.09090909	Shared protein domains PFAM
MT4	MT1F	0.09090909	Shared protein domains PFAM
MT1X	MT1A	0.09090909	Shared protein domains PFAM
MT1X	MT1B	0.09090909	Shared protein domains PFAM

MT1X	MT1M	0.09090909	Shared protein domains PFAM
MT1X	MT1G	0.09090909	Shared protein domains PFAM
MT1X	MT1E	0.09090909	Shared protein domains PFAM
MT1X	MT1H	0.09090909	Shared protein domains PFAM
MT1X	MT1F	0.09090909	Shared protein domains PFAM
MT1X	MT4	0.09090909	Shared protein domains PFAM
MT3	MT1A	0.09090909	Shared protein domains PFAM
MT3	MT1B	0.09090909	Shared protein domains PFAM
MT3	MT1M	0.09090909	Shared protein domains PFAM
MT3	MT1G	0.09090909	Shared protein domains PFAM
MT3	MT1E	0.09090909	Shared protein domains PFAM
MT3	MT1H	0.09090909	Shared protein domains PFAM
MT3	MT1F	0.09090909	Shared protein domains PFAM
MT3	MT4	0.09090909	Shared protein domains PFAM
MT3	MT1X	0.09090909	Shared protein domains PFAM
MT2A	MT1A	0.09090909	Shared protein domains PFAM
MT2A	MT1B	0.09090909	Shared protein domains PFAM
MT2A	MT1M	0.09090909	Shared protein domains PFAM
MT2A	MT1G	0.09090909	Shared protein domains PFAM
MT2A	MT1E	0.09090909	Shared protein domains PFAM
MT2A	MT1H	0.09090909	Shared protein domains PFAM
MT2A	MT1F	0.09090909	Shared protein domains PFAM
MT2A	MT4	0.09090909	Shared protein domains PFAM
MT2A	MT1X	0.09090909	Shared protein domains PFAM
MT2A	MT3	0.09090909	Shared protein domains PFAM
MT1HL1	MT1A	0.09090909	Shared protein domains PFAM
MT1HL1	MT1B	0.09090909	Shared protein domains PFAM
MT1HL1	MT1M	0.09090909	Shared protein domains PFAM
MT1HL1	MT1G	0.09090909	Shared protein domains PFAM
MT1HL1	MT1E	0.09090909	Shared protein domains PFAM
MT1HL1	MT1H	0.09090909	Shared protein domains PFAM
MT1HL1	MT1F	0.09090909	Shared protein domains PFAM
MT1HL1	MT4	0.09090909	Shared protein domains PFAM
MT1HL1	MT1X	0.09090909	Shared protein domains PFAM
MT1HL1	MT3	0.09090909	Shared protein domains PFAM
MT1HL1	MT2A	0.09090909	Shared protein domains PFAM
